# A materials informatics driven fine-tuning of triazine-based electron-transport layer for organic light-emitting devices

**DOI:** 10.1038/s41598-024-54473-3

**Published:** 2024-02-22

**Authors:** Kosuke Sato, Kazuki Hattori, Fuminari Uehara, Tomoko Kitaguni, Toshiki Nishiura, Takuya Yamagata, Keisuke Nomura, Naoki Matsumoto, Tsuyoshi Tanaka, Hidenori Aihara

**Affiliations:** 1 Sagami Chemical Research Institute, Ayase, Kanagawa 252-1193 Japan; 2 Tokyo Research Center, Organic Materials Research Laboratory, Tosoh Corporation, Ayase, Kanagawa 252-1123 Japan

**Keywords:** Electronic devices, Electronic materials

## Abstract

Materials informatics in the development of organic light-emitting diode (OLED) related materials have been performed and exhibited the effectiveness for finding promising compounds with a desired property. However, the molecular structure optimization of the promising compounds through the conventional approach, namely the fine-tuning of molecules, still involves a significant amount of trial and error. This is because it is challenging to endow a single molecule with all the properties required for practical applications. The present work focused on fine-tuning triazine-based electron-transport materials using machine learning (ML) techniques. The prediction models based on localized datasets containing only triazine derivatives showed high prediction accuracy. The descriptors from density functional theory calculations enhanced the prediction of the glass transition temperature. The proposed multistep virtual screening approach extracted the promising triazine derivatives with the coexistence of higher electron mobility and glass transition temperature. Nine selected triazine compounds from 3,670,000 of the initial search space were synthesized and used as the electron transport layer for practical OLED devices. Their observed properties matched the predicted properties, and they enhanced the current efficiency and lifetime of the device. This paper provides a successful model for the ML assisted fine-tuning that effectively accelerates the development of practical materials.

## Introduction

The data-science driven development of novel functional materials has attracted attention and has been promoted for its cost-effectiveness and exhaustiveness^1–4^. However, the difficulty of the systematic data accumulation and the localization of informatics projects in organic electronics have limited the employment of data-driven approaches^5,6^. Furthermore, data-driven approaches for the molecular fine-tuning is still under development. Herein, we report a model case of a materials informatics (MI) project which focuses on achieving the coexistence of multiple superior properties in a single molecule. By using the step-by-step screening method that synergistically combines machine learning (ML) and scientists’ knowledge, we have successfully fine-tuned electron-transport compounds for use in organic light-emitting diode (OLED) devices.

Recently, MI strategies have been studied in the field of organic electronics^5–8^. These strategies have enabled the development of novel molecules with desired properties through data science-assisted structure-property relationship prediction. Computational virtual screening methods have been used to explore novel organic semiconductors^9–11^, fluorescent molecules^12,13^, and emitter molecules for OLEDs^14–17^. In a previous study, Aspuru-Guzik et al. reported a high throughput screening method that used an ML prediction model based on large datasets between molecular structures and density functional theory (DFT) calculations to calculate rate constants of reverse intersystem crossing^17^. Although the sophisticated DFT calculations can contribute to effective property prediction, simulations in long-range and dynamic molecular behavior require significant computational resources. Especially, the carrier mobility and glass transition behavior can be simulated; however they require large-scale molecular dynamics calculations^18–22^. Therefore, it is difficult to achieve the high-throughput prediction of the essential properties of the carrier transport materials, next to the light emitting layer in OLED devices^8^. The fact implies that traditional supervised ML is still a powerful prediction tool for exploring the carrier transport materials. In the present work, the prediction of electron mobility and glass transition temperature was achieved via typical ML methods using hundreds of experimental datasets. The present prediction models, enhanced by low-level DFT calculations, enabled virtual screening to find molecules with versatile superior properties.

OLEDs are practical and promising technologies for manufacturing vivid and colorful displays used in daily life^23–26^. To enhance the external quantum efficiency and driving voltage characteristics, the development of electron-transport materials is crucial^25,26^. Among these materials, 1,3,5-triazine derivatives play an important role in the component materials of OLED devices^27–32^. Their electron negativity and planar nature lead to the high electron mobility and durability in practical usage as an electron-transport layer (ETL). In the previous works, the molecular design of triazine derivatives was studied to find compounds with higher electron mobility^29–32^. However, the general strategy to enhance electron mobility is yet to be fully understood because complicated intermolecular interactions and molecular geometry affect carrier transport. Additionally, a higher glass transition temperature is strongly required for long-term durability^33–35^ even though the thermodynamic behavior of triazine derivatives remains unclear. Furthermore, practical device fabrication requires appropriate conduction band levels and applicability to the vapor deposition method^25,26,35^. The balancing these properties often presents a challenge beyond human intelligence. If ML-based techniques can realize the high-throughput predictions of the required properties, the exploration of novel materials will be dramatically accelerated.

**Figure 1:**
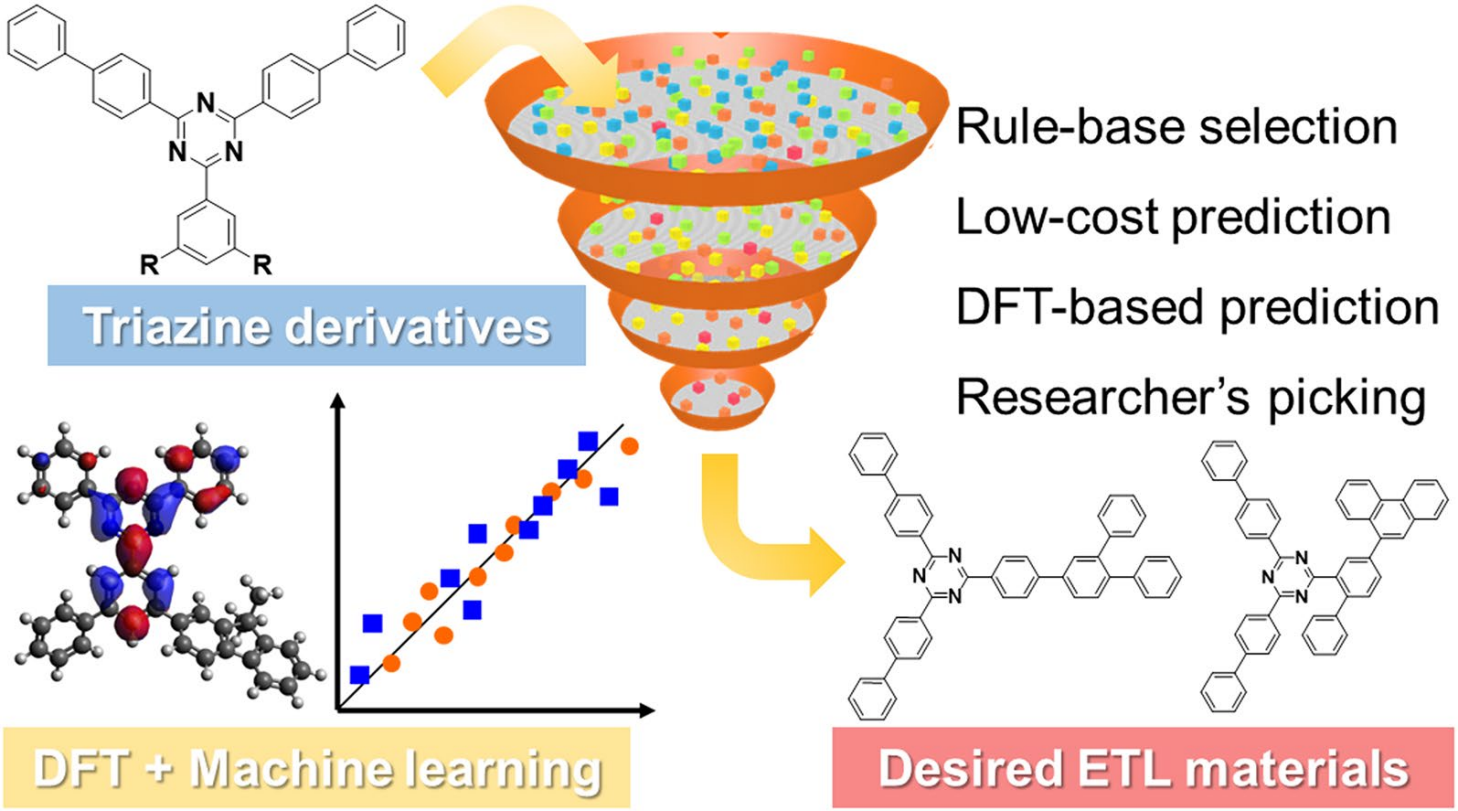
Schematic illustration of the proposed virtual screening.

Here, we present a model case of data-driven fine-tuning to discover novel triazine derivatives. We propose a hierarchical combination, including empirical knowledge from scientists, DFT calculations, and ML to select compounds (Fig. 1). The resulting triazine derivatives showed enhanced properties as the ETL and functioned as a practical component for highly efficient and durable OLED devices.

## Methods

### Device fabrication

All reagents were used after sublimation purification. A glass substrate on which transparent indium-tin oxide (ITO) electrode was printed as a stripe pattern was prepared. The substrate was washed with isopropyl alcohol and then its surface was treated by irradiation of ultraviolet rays. The area of the test devices was 4 mm^2^ (2 mm × 2 mm). The glass substrate was placed in a vacuum deposition chamber, and the inner pressure was reduced to 1.0 × 10^−4^ Pa. Each layer was formed by vacuum deposition. The detailed procedure and molecular structures of the device components are described in the supporting information (SI).

### Characterization

The thermodynamic property of the triazine compounds was measured by differential scanning calorimetry (DSC, Hitachi High-Tech, DSC7020). Typically, 5 mg of the sample loaded in sealed aluminum pan was maintained at 360 °C for 5 min. Then, the melted sample was cooled by putting it on dry ice to obtain the glass-state sample. The DSC chart of heating process was measured at a rate of 10 °C min^−1^ rate in range from 40 °C to 350°C. The fabricated electron-only-device (EOD) and the OLED device were tested by an electrometer (Keithley 2400). Luminous properties of the OLED device were evaluated by applying a direct current and using a luminance meter (Topcon Technohouse, BM-9). The current density and emission intensity were recorded at various terminal voltages. To test the long-term durability, the current density was kept to the initial value corresponding to 1000 cd m^−2^ of the emission intensity.

### Data handling and machine learning process

The analysis of the V–J curve based on the space-charge-limited current (SCLC) model^36^ and the time-dependent degradation analysis of the OLED devices were automated using Visual Basic for Applications code running on Excel interface. The sum of errors between the experimental and the simulated values was minimized by selecting of unknown variables. The generalized reduced gradient method (nonlinear) was used for the optimization algorithm. The data handling and machine learning to predict structure-property relationships were performed by using PyCaret library running on Python 3.6.1 environment^37^. The descriptors of the molecular structures were generated by using Mordred^38^. To obtain three-dimensional (3D) optimized structures and lowest unoccupied molecular orbital (LUMO) levels, DFT calculation was performed on B3LYP/6-31G(d) level theory.

## Results and discussion

### Training dataset of triazine compounds

**Figure 2:**
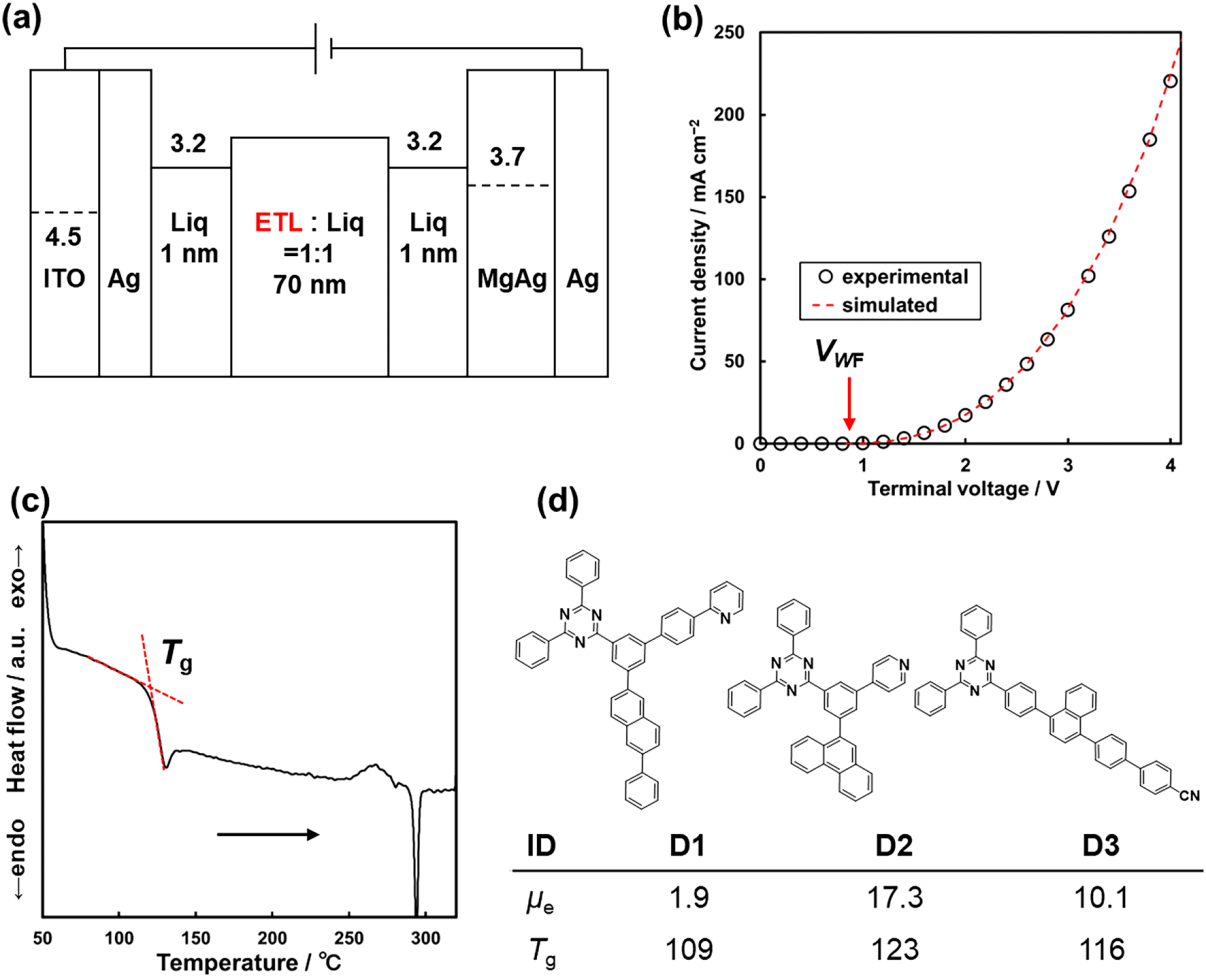
(**a**) Schematic architecture of the electron only device (EOD) used in the present study. (**b**) V–J curve of the EOD including **D3**. The *V*_WF_ was estimated from the fitting curve based on SCLC model (red hashed line). (**c**) DSC chart of **D3**. (**d**) Examples of the initial dataset comprising of the various triazine derivatives.

The initial structure-property datasets used in this study consisted of electron mobility (*μ*_e_) data (*N* = 202), that were measured by using EOD, and glass transition temperature (*T*_g_) data (*N* = 551) of various triazine derivatives. The initial datasets were shared from TOSOH Corporation. In the EOD fabrication, the triazine derivatives were co-deposited with 8-quinolinolato lithium (**Liq**) for smooth carrier injection (Fig. 2a). Since the ETL in the device contained only 50 wt% of the triazine compounds, the observed *μ*_e_ were lower than that measured under typical conditions. Therefore, the mobility values in the dataset did not represent those of the pure compound but the mixture composed of ETL compound and **Liq**. The *μ*_e_ was calculated from the V–J curve under the assumption of the SCLC model Eq. (1)^36^ (Fig. 2b). The following variables were used as constants in the fitting: the temperature (*T*), the thickness of the active layer (*L*), and relative permittivity (*ε*) were 25°C, 70 nm, and 3.6 for all the compounds, respectively.

1 $$J = \frac{9}{8}\mu \varepsilon \varepsilon_{0} \frac{{\left( {V - V_{WF} } \right)^{2} }}{{L^{3} }}\exp \left( {\frac{0.891}{{kT}}\left( {\frac{{e^{3} \left( {V - V_{WF} } \right)}}{{\pi \varepsilon \varepsilon_{0} L}}} \right)^{0.5} } \right)$$

By optimizing two variables, *μ*_e_ and the voltage loss corresponding to the working function (*V*_WF_), the error between the experimental and simulated chart was minimized. When multiple EOD data were measured for the same compound, the average value was used in the final dataset. The *μ*_e_ values ranged from 0.1 to 60 ×10^−6^ cm^2^ V^−1^ s^−1^ (Fig. S2). The accuracy of the proposed *μ*_e_ measurement procedure was calculated from the same device setup (using **D3** as ETL material, *N* = 32). The mean absolute error (MAE) and coefficient of variation (C_V_) is ±3.3 × 10^−6^ cm^2^ V^−1^ s^−1^ and 32%, respectively. The accuracy of the EOD measurement was regarded as the target level of the prediction model.

The *T*_g_ data of the compounds were measured by DSC. To obtain the glass state, the samples were placed on sealed aluminum pan and heated above their melting point before being rapidly cooled. In the typical DSC chart of the heating process, a baseline shift and endothermic peak were observed (Fig. 2c). The former corresponds to the *T*_g_ while the latter corresponds to the melting point. The *T*_g_ values distributed between 80 °C and 180 °C (Fig. S2). For example, the training dataset were composed of triphenyl triazine derivatives with various terminal groups such as phenyl, naphthyl, phenanthryl and pyridyl groups (Fig. 2d).

### Prediction models

**Figure 3:**
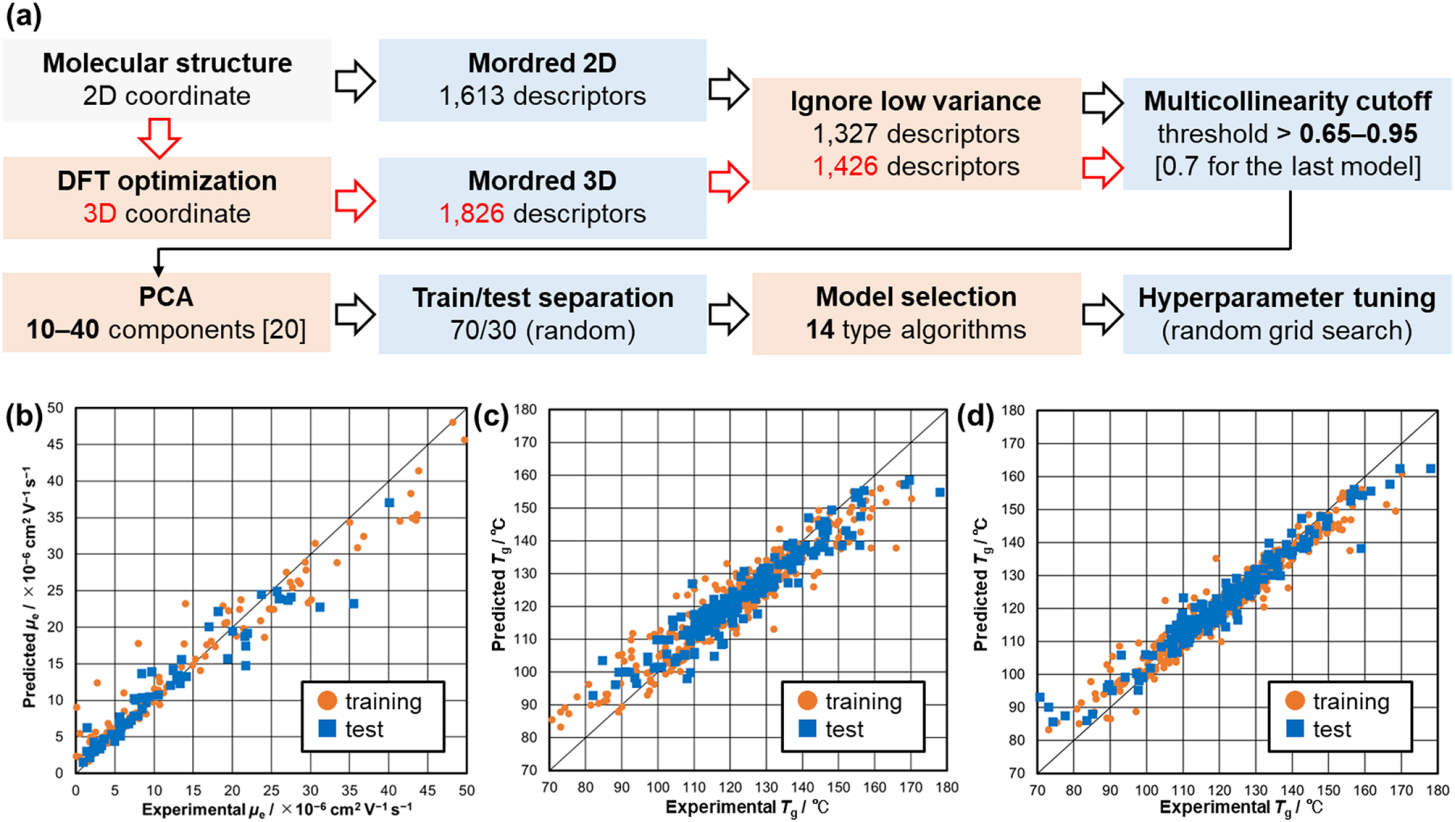
(**a**) Schematic pipeline representing the descriptor generation and prediction model construction. (**b**–**d**) Predicted-experimental plots of the machine learning models. (**b**) Random forest model for *µ*_e_ (**c**) Extra tree model for *T*_g_(2D) (**d**) Extra tree model for *T*_g_(3D).

To construct supervised ML models, Mordred was used to calculate the descriptors of the compounds^38^. Mordred is a descriptor-calculation software widely used in cheminformatics research; it can generate 1,613 descriptors from 2D molecular structure. The descriptors comprised the numbers from counting atoms/bonds/rings, graph-based indices, and numerous autocorrections, etc. Several descriptors were removed because of zero-distribution and multicollinearity (Fig. 3a). As a result of the initial cleaning, the numbers of the descriptors were narrowed to a range of 50–90, depending on the threshold values. After tuning to improve the accuracy of the prediction model, the correlation coefficient cutoff threshold was set to 0.7 during preprocessing. The ML model training was performed by using the PyCaret library^37^, which contains powerful and low-code functions to perform typical ML methods. The initial dataset was separated into training and test data at a ratio of 70:30 and prediction models using various algorithms were trained with default values of hyperparameters. Thereafter, we selected five expecting algorithms based on the initial MAE scores of the test data (Fig. S3). The hyperparameters of the training models based on the selected algorithms were optimized by 5-fold cross-validation. The best prediction model was determined by considering predicted vs. experimental plot of each model and the gap between MAEs of the training and test data. The prediction accuracy was primarily evaluated by the MAE score and compared to the corresponding measurement accuracy.

For the prediction of *μ*_e_, four linear regression models and ten tree-based models were adopted based on the PyCaret library (Fig. S4). The number of descriptors was reduced to 20 by principal component analysis (PCA) method to address the challenge of the small amount of the training data, after trying several values. The cumulative proportion of the components was 0.84 in the condition. Through the tuning process of the hyperparameters, the random forest model was obtained with an MAE score of 3.4 × 10^−6^ cm^2^ V^−1^s^−1^ (Table 1 and Fig. 3b). Even though the test data plots in the high-mobility region were scattered in the predicted vs. experimental plot, the predicted and experimental *μ*_e_ were consistent in the range of 1–30 × 10^−6^ cm^2^ V^−1^ s^−1^. Since the MAE value is comparable to the accuracy of the *μ*_e_ measurement, we used the resulting model for the virtual screening. The goal of this work is to discover practical materials with enhanced properties through fine-tuning the existing materials. Thus, the superior performance for the existing triazine derivatives included in the initial datasets was required to accurately predict the properties of related triazine derivatives, which are among the neighboring search spaces.

**Table 1:** Characteristic values of the machine learning models.

	Data size	Algorithm	Data accuracy	MAE (training)	MAE (test)	R^2^
*μ*_e_/cm^2^ V^−1^s^−1^	202	RandomForest	± 3.3 × 10^−6^	3.0 × 10^−6^	3.4 × 10^−6^	0.87
*T*_g_(2D)/ °C	551	ExtraTree	± 3.0	5.5	5.8	0.88
*T*_g_(3D)/ °C	551	ExtraTree	± 3.0	3.2	3.4	0.93

ML-based *T*_g_ prediction was performed by the same procedure for the *μ*_e_ prediction (Fig. 3a). The number of descriptors was reduced to 20 by PCA method, for the same reason as the *μ*_e_ prediction. After optimizing the hyperparameters on the Extra tree algorithm, the training model based on the 2D descriptors, *T*_g_(2D) model, showed an MAE of 5.8 °C (Fig. 3c). While the value did not reach the measurement accuracy, we added descriptors that could be calculated from the 3D molecular structure. The optimized structures were obtained from DFT calculations on B3LYP/6-31G(d) level theory. Mordred can generate 1,826 descriptors from the 3D molecular structure. The number of the features was reduced using the same procedure. The remaining descriptors contained partial surface area descriptors, geometric radius descriptors, and molecular representations of structures based on electronic diffraction (3D-MoRSE) descriptors. The 3D descriptors derived from the DFT-assisted optimized structures improved the prediction accuracy. The MAE score reduced from 5.8 °C to 3.4 °C (Fig. 3d and Table 1). Both the *T*_g_(2D) and *T*_g_(3D) models were used for the screening, considering the trade-off between the computational cost and the prediction accuracy.

The ML model for *T*_g_ prediction exhibited the best performance among that for low-molecular weight organic compounds related to OLED. While several earlier works reported a quantitative structure-property relationship and ML models for *T*_g_ prediction, their accuracy was lower than that of the present work (Table S1)^8,16,39–41^. The use of ML techniques is a simple and effective way to improve the accuracy of the *T*_g_ prediction. According to the difference between predicted the *T*_g_(2D) and *T*_g_(3D) value, 3D structure of the compounds contributes to the enhanced prediction accuracy. The superior prediction accuracy of the *T*_g_(3D) model was confirmed by the comparison based on the same train/test separation (Table S1). Despite the low-level theory used in the DFT calculations, the 3D descriptors can still play important roles in *T*_g_ prediction. Our intention here is that the low-cost DFT calculations have great potentials to produce the effective descriptors. With a small target search space, it is not reasonable to hesitate to perform DFT calculations, considering the trade-off between the cost and their usefulness.

### Efficient discovery of new compounds by virtual screening

**Figure 4:**
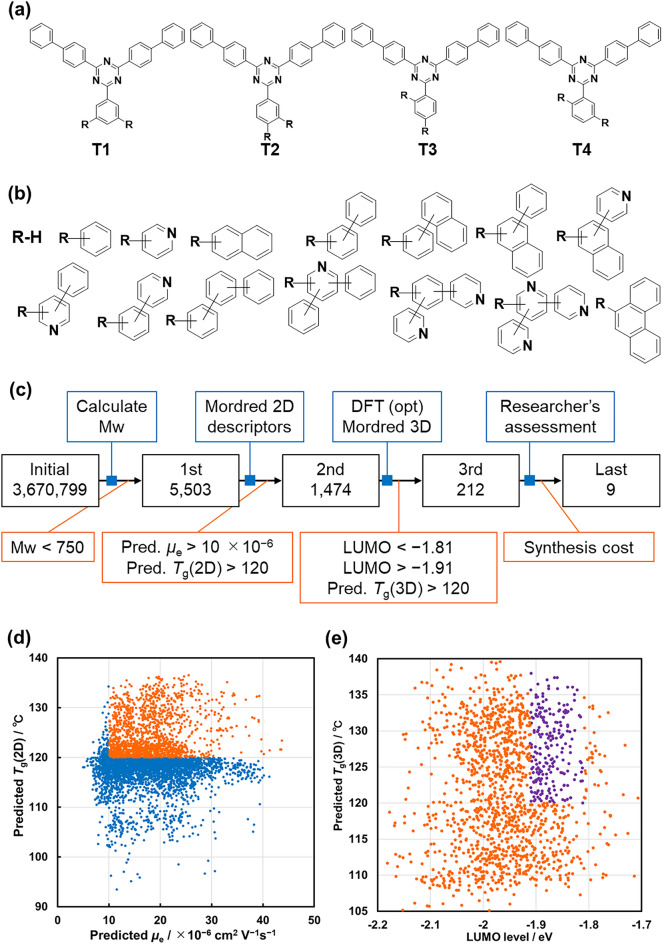
(**a**) Core triazine structures for building the virtual molecular library. R indicates attachment points for R-group enumeration. (**b**) Schematic representation of the terminal groups for building the virtual molecular library. R indicates attachment points for the core triazine structure. (**c**) Attrition diagram of the screening process. (**d**) Relationships between the predicted *μ*_e_ and the predicted *T*_g_(2D). Compounds with lower scores (blue) were dropped, and others (orange) were passed. (**e**) Relationships between the calculated LUMO level and the predicted *T*_g_(3D). Compounds with lower score (orange) was dropped and others (purple) were passed.

The proposed multistep screening consists of four steps (Fig. 4a). Since we focused on the coexistence of the sublimability, high *μ*_e_, upper *T*_g_, and appropriate LUMO level, the step-by-step filtering of the candidates was applied from the low-cost property prediction to the high-cost one. After that, among the promising candidates, those with low synthesis cost and high novelty were manually selected.

The virtual compound library was created by combining core triazine structures and terminal functional groups^42^. The 4 types of the core triazine structures containing bis(4-biphenyl) triazine moiety were selected (Fig. 4b). The 1,2,3-substitution type structures were not considered because of their large steric hindrance. The aromatic groups consisting of up to 18 carbon atoms were used as the terminal groups. They were curated from a partial structure composing the initial datasets. The pyridyl groups were included to ensure the diversity of LUMO levels and intermolecular interactions. The anthracenyl, pyrenyl, and benzanthracenyl groups were excluded because they would cause unwanted fluorescence in OLED devices. The number of the generated terminal groups was 1,087. The entire virtual compound library contained almost 3,670,000 triazine derivatives.

On the first step of the screening, the search space was simply reduced by the upper limit of the molecular weight. In our experimental knowledge, the sublimation temperature of the triazine derivatives with a molecular mass of 750 g/mol and more are expected to exceed 370 ℃, which is higher than their thermal decomposition temperature. Furthermore, lower sublimation temperatures are required in a mass production process compared to a lab-scale synthesis to shorten the process. The first step screening by the molecular weight filter extracted 5,503 compounds from the whole search space.

On the second step, the *μ*_e_ and *T*_g_(2D) predictions were performed by the ML models. The selected threshold values of the predicted *μ*_e_ and *T*_g_(2D) were 10 × 10^−6^ cm^2^ V^−1^s^−1^ and 120 °C, respectively. The threshold value for the *μ*_e_ was derived from the top 40 percentile values in the initial experimental datasets and that of the *T*_g_(2D) was a required value owing to the sealing process on the device fabrication. The ML-based screening revealed that 2000 triazine derivatives were expected to acquire efficient electron-transport properties, of which about 1,474 compounds (0.03% of the whole search space) showed promising glass-state stability (Fig. 4c,d). Then, the optimized coordinate of the resulted candidates was calculated by the DFT method on the B3LYP/6-31G(d) level theory by Gaussian 16.

On the third step, the number of compounds was narrowed by the DFT based feature and *T*_g_(3D) prediction (Fig. 4e). We screened the candidates based on their LUMO level since the LUMO level close to that of the hole-blocking layer was required to reduce the interface resistance. The cutoff value of the LUMO level was upper −1.91 eV due to −1.81 eV for the hole blocking layer material (Fig. S1). In addition, 340 compounds were excluded because of their low predicted *T*_g_(3D), although the predicted *T*_g_(2D) of the candidates in this step were higher than 120 °C. The difference between the *T*_g_(2D) and *T*_g_(3D) represents the effectiveness of DFT-based 3D descriptors. Although 1,474 candidates on the second screening step were predicted to exhibit an *μ*_e_ and *T*_g_ of more than 10 × 10^−6^ cm^2^ V^−1^ s^−1^ of *μ*_e_ and 120 °C of *T*_g_, the number of candidates was narrowed down to 212 by screening using the LUMO levels and *T*_g_(3D), which reduced the number by 76%.

On the final step, we employed human-guided decision-making to reduce the search space. The 212 most promising compounds were listed up and shared this list with relevant stakeholders. Domain experts assessed these candidates based on several criteria, including synthesis difficulty, synthesis cost, structural diversity, and novelty. In the decision making, the compounds that required expensive reagents, such as organic tin reagents for the Stille coupling reaction and highly substituted pyridines, were removed because the goal of our project is to explore practical compounds that can be produced at low cost. The compounds containing the high-cost moiety were removed through the assessment. The examples of excluded molecules and the reasons not to be selected were described in the SI (Table S4).

Finally, 9 molecules were selected with the accomplished consensus for synthesis and characterization as ETL materials.

### Synthesis and properties of the novel compounds

The selected 9 compounds were synthesized (Schemes S1–S9, Figs. S5–S6 in the SI). The obtained *μ*_e_ and *T*_g_ data were compared to the predicted ones (Table 2). Although several compounds, (**T3-3317**, **T1-4799** and **T1-5248**) had a gap between the predicted and experimental data, the other 6 compounds showed good consistency with predicted and experimental *μ*_e_. The *T*_g_(3D) values also showed similar values to the experimental *T*_g_, except for some compounds (**T2-6104**, **T2-6970**, and **T4-442**). The comparison of the predicted and experimental values demonstrates the usefulness of the ML models in the application of unknown compounds.

**Table 2:** Molecular structures of the synthesized triazine compounds, their calculated LUMO level, predicted *μ*_e_, experimental *μ*_e_, *T*_g_(2D), *T*_g_(3D), and experimental *T*_g_.

Structure					
ID	T2-7668	T2-6104	T2-6970	T2-7191	
LUMO level/eV	− 1.88	− 1.86	− 1.86	− 1.90	
Predicted *μ*_e_ × 10^−6^/cm^2^ V^−1^ s^−1^	20.8	19.7	22.8	13.3	
Experimental *μ*_e_ × 10^−6^/cm^2^ V^−1^ s^−1^	26.0	19.6	34.9	17.5	
Predicted *T*_g_(2D)/ °C	122	130	127	121	
Predicted *T*_g_(3D)/ °C	121	122	137	129	
Experimental *T*_g_/ °C	120	129	158	132	

Structure					
ID	T4-442	T4-2766	T3-3317	T1-4799	T1-5248
LUMO level/eV	− 1.85	− 1.85	− 1.86	− 1.84	− 1.91
Predicted *μ*_e_ × 10^−6^/cm^2^ V^−1^ s^−1^	10.6	12.6	11.3	13.9	14.8
Experimental *μ*_e_ × 10^−6^/cm^2^ V^−1^ s^−1^	14.0	16.5	24.7	6.4	4.3
Predicted *T*_g_(2D)/ °C	132	127	125	126	128
Predicted *T*_g_(3D)/ °C	130	121	122	121	122
Experimental *T*_g_/ °C	137	116	116	120	118

Our intention here is the improved efficiency in the discovery of new practical compounds. The efficiency ratio, i.e. superior 5 compounds per synthesized 9 compounds, was considered that the proposed screening method can find compounds with desired properties with the 56% expectation. In the initial experimental datasets used to build the ML model, the 54 compounds in whole 202 compounds exhibited both upper than 10 × 10^−6^ cm^2^ V^−1^ s^−1^ of *μ*_e_ and 120 °C of *T*_g_. The ratio, only 27%, can be regarded as the expectation value of the human driving development without informatics-based techniques. Therefore, the acceleration of the fine-tuning was achieved by the proposed screening method. Although the local and limited data accumulated in commercial companies provide domain-specific machine learning models, that can contribute to detailed molecular design on practical development.

**Figure 5:**
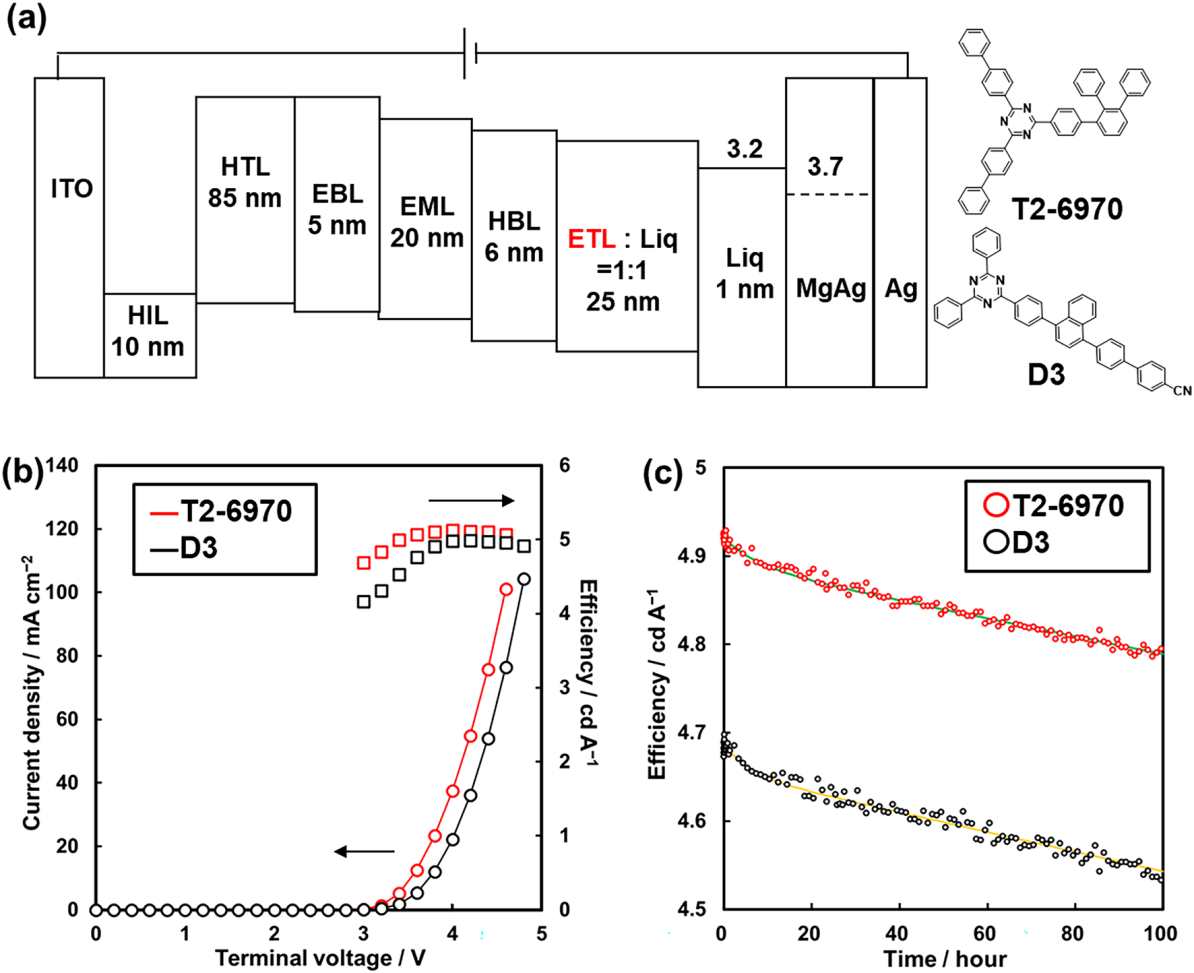
(**a**) Schematic architecture of OLED device used in the present study. (**b**) Relationship of terminal voltage and current density (circles), and current efficiency (squares) on OLED devices. (**c**) Time-efficiency plot of OLED devices on constant current mode maintaining the initial current density. The initial current density was corresponding to 1000 cd m^−2^ of the emission intensity. The simulated lines, green for and yellow for **T2-6970** and **D3**, respectively, were calculated from the fitting of the time-dependent degradation model^34^.

**T2-6970**, exhibiting the highest *µ*_e_, the highest *T*_g_, and a suitable LUMO level, was applied to the OLED device, and its properties as ETL material were evaluated (Fig. 5a, S4, and Table S3). We selected **D3** as a reference ETL compound for the property comparison because **D3** could be regarded as a representative ETL material and had been used in the practical OLED devices^43^. Compared to **D3**, the current density of the **T2-6970** device increased at the same terminal voltage (Fig. 5b). The superior electron mobility of **T2-6970** resulted in lower terminal voltage of the device. The maximum current efficiency of the **T2-6970** device was higher than that of the **D3** device. The improvement should originate from the change in the carrier balance factor since the higher electron mobility of **T2-6970** leads the recombination region to near the hole-transporting layer. The long-term durability was evaluated by monitoring the efficiency degradation over time (Fig. 5c). The efficiency degradation rate was analyzed by fitting the time dependence plot with the dual time-constant model Eq. (2)^35^. The observed current efficiency over time (*E*) was indicated by the short and long-term efficiency degradation: *E*_0_ is the initial efficiency, *t* is time, *a* is ratio of short/long-term factors, *τ*_S_ is a time constant, and *τ*_L_ is another time constant.

2 $$E = E_{0} \left( {a\exp \left( {\frac{t}{{\tau_{S} }}} \right) + \left( {1 - a} \right)\exp \left( {\frac{t}{{\tau_{L} }}} \right)} \right).$$

Three variables, *a*, *τ*_S_, and *τ*_L_ were optimized to minimize the error between the experimental and simulated efficiency values. The long-term time constant, *τ*_L_, was 4,750 and 4,114 hours for the **T2-6970** and **D3** devices, respectively. The efficiency degradation rate seems to be suppressed due to the higher *T*_g_ of **T2-6970**, which is intended to prevent undesired crystallization in the device. Since the threshold value in the screening was determined according to practical use, **T2-6970** is a very promising material with suitable properties for multiple aspects. Whereas the existing triazine compounds with large π-conjugated LUMO and appropriate LUMO levels have been explored to enhance electron-transport properties, we successfully found a fine-tuned molecular structure through a hierarchical virtual screening approach. Moreover, high-performance ETL materials can be efficiently examined using the proposed methods as shown in Fig. 1.

## Conclusion

A virtual screening method was applied to find novel triazine derivatives for ETL materials in OLEDs. By using a typical ML method, the prediction accuracy was comparable to the measurement accuracy (an *µ*_e_ and *T*_g_ of 3.4 × 10^−6^ cm^2^ V^−1^ s^−1^ and 3.4 °C, respectively). The descriptors from DFT calculations enhanced the prediction of *T*_g_. To fine-tune and obtain triazine derivatives with suitable properties for multiple aspects, a screening scheme combining the predicting models and experimental knowledge was designed. The promising triazine derivatives with the coexistence of higher *µ*_e_ and *T*_g_ were successfully extracted from the proposed virtual compound library. The resulting fine-tuned compound, **T2-6970**, exhibited high current efficiency and a long lifetime in the practical OLED device. The present work proposes that the ML-assisted hierarchical virtual screening method is useful for fine-tuning of molecular-based materials. Further approaches to analyze the datasets, such as the clustering techniques to extract effective molecular structures, are ongoing to discover future molecular designs.

### Supplementary Information

Supplementary Information.

## Data Availability

The synthesized compounds and property data in this study are included in this article. The initial datasets for the prediction models were managed in Tosoh Corporation.
