# Supplementary material for: A materials informatics driven fine-tuning of triazine-based electron-transport layer for organic light-emitting devices
Source: Sci Rep. 2024 Feb 22;14:4336. doi: 10.1038/s41598-024-54473-3 (PMC10881559; doi:10.1038/s41598-024-54473-3)
Supplement: Supplementary file 1 — Supplementary Information. [file 41598_2024_54473_MOESM1_ESM.pdf]

Supplementary Information

A Materials Informatics Driven Fine-Tuning of Triazine-Based Electron-Transport Layer for Organic Light-Emitting Devices

Kosuke Sato*, Kazuki Hattori, Fuminari Uehara, Tomoko Kitaguni, Toshiki Nishiura, Takuya Yamagata, Keisuke Nomura, Naoki Matsumoto, Tsuyoshi Tanaka, Hidenori Aihara

Sagami Chemical Research Institute

2743-1 Hayakawa, Ayase, Kanagawa 252-1193, Japan

E-mail: sato.k.cx@m.titech.ac.jp

Contents

Detailed procedure and materials in device fabrication (Figure S1)	P. S2
Synthesis methods (Scheme S1-S9)	P. S5
Histograms of training datasets (Figure S2)	P. S14
Initial screenings of machine learning models on PyCaret (Figure S3)	P. S15
Comparison of T_g prediction models (Table S1)	P. S16
Examples of the structure-predicted properties in the last screening step (Table S2)	P. S17
Luminescence spectra and other properties of the fabricated OLED devices (Figure S4)	P. S18
Raw NMR and LC-MS data of the synthesized compounds (Figure S5, S6)	P. S19
Supporting references	P. S33

Detailed procedures and materials in device fabrication

Characterization of the synthesized compounds

Nuclear magnetic resonance (NMR) charts of ^{1}H and ^{13}C were obtained by NMR equipment (Bruker, Ascend 400 MHz for ^{1}H and 100 MHz for ^{13}C). The samples were dissolved in $CDCl_3$. Liquid chromatography - mass spectroscopy (LC-MS) measurements was performed by integrated LC-MS equipment (Shimazu, LC-MS 2020). The chromatograms were obtained by following conditions: ZORBAX Eclipse XDB-C18 (250 mm) for main column, methanol/THF = 90/10 (v/v) for eluent, 40 °C for column oven temperature, 1.0 ml/min of feeding speed, and absorption at 254 nm monitored by photodetector. The MS profiles corresponding to the main peak in the chromatograms were obtained with electrospray ionization (ESI).

Device fabrication procedure

All reagents were used after sublimation purification. A glass substrate with a transparent indium-tin oxide (ITO) electrode was prepared, which had a stripe pattern comprised of ITO film with 110 nm thickness and 2 mm width. The substrate was washed with isopropyl alcohol and then surface-treated by irradiation of ultraviolet rays. The area of the test device is 4 mm^2 (2 mm × 2 mm) square, that was patterned by metal mask. The thickness of each layer was measured by film thickness gauge (DEKTAK, Bruker). The glass substrate was placed in a vacuum deposition chamber, and the inner pressure was reduced to 1.0×10^{-4} Pa. Each layer was formed by vacuum deposition as follows.

For EOD (Figure 2a),

Anode: Ag was deposited for 20 nm with 0.2 nm sec^{-1} rate.

Electron injection layer: **Liq** was deposited for 1 nm.

Electron transport layer: the mixture containing 50 wt% of the triazine derivative and 50 wt% of **Liq** was co-deposited for 70 nm with 0.15 nm sec^{-1} rate.

Electron injection layer: **Liq** was deposited for 1 nm.

Cathode: After a striped metal mask was arranged to be orthogonal to the ITO stripe, Mg/Ag alloy (10/1, wt/wt) was deposited for 80 nm with 0.5 nm sec^{-1} rate. Then, Ag was deposited for 20 nm with 0.2 nm sec^{-1} rate.

For OLED (Figure S1),

Hole injection layer: the mixture containing 50 wt% of **HIL** and 50 wt% of **HTL** was co-deposited for 10 nm with 0.15 nm sec^{-1} rate.

Hole transport layer: **HTL** was deposited for 10 nm with 0.15 nm sec^{-1} rate.

Electron blocking layer: **EBL** was deposited for 10 nm with 0.15 nm sec^{-1} rate.

Emitting layer: the mixture containing 5 wt% of **Emitter** and 95 wt% of **Host** was co-deposited for 25 nm with 0.18 nm sec^{-1} rate.

Hole blocking layer: **HBL** was deposited for 5 nm with 0.15 nm sec^{-1} rate.

Electron transport layer: the mixture containing 50 wt% of the triazine derivatives and 50 wt% **Liq** was co-deposited for 25 nm with 0.15 nm sec^{-1} rate.

Electron injection layer: Yb was deposited for 2 nm with 0.10 nm sec^{-1} rate.

Cathode: After a striped metal mask was arranged to be orthogonal to the ITO stripe, Mg/Ag alloy (10/1, wt/wt) was deposited for 80 nm with 0.5 nm sec^{-1} rate. Then, Ag was deposited for 20 nm with 0.2 nm sec^{-1} rate.

After the deposition, the obtained assembly of multi-layers was encapsulated with a glass cap and ultraviolet ray-curable epoxy resin (purchased from Nagase Chemtex). The encapsulation was conducted in a nitrogen atmosphere having an oxygen-and-moisture content of below 1 ppm within a glove box.

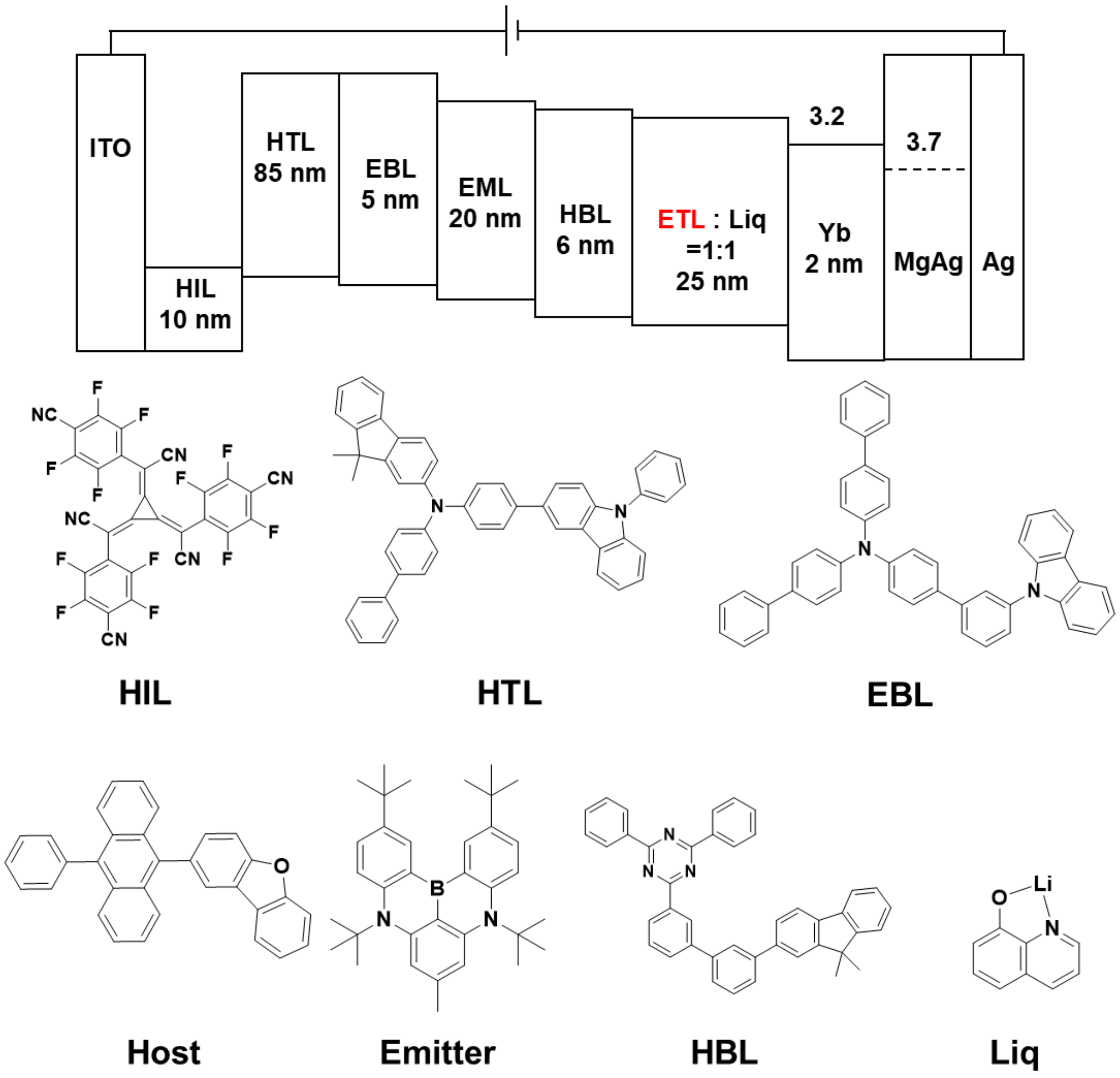

Figure S1 Materials for blue light emission OLED used in the present work.

Synthesis methods (Scheme S1-S9)

Scheme S1 synthesis of **T2-7668**

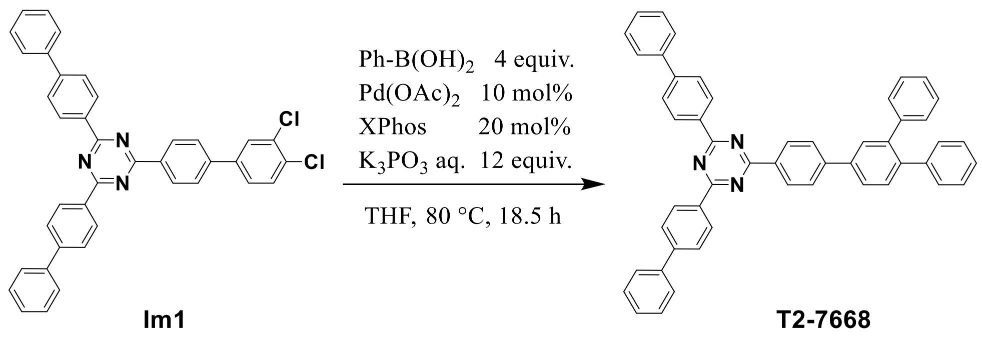

T2-7668. 2,4-Bis(biphenyl-4-yl)-6-(3',4'-dichloro-bephenyl-4-yl)-1,3,5-triazine (**Im1**) was synthesized via known method reported in the previous work.[s1] Under argon atmosphere, **Im1** (9.90 g, 16 mmol), phenyl boronic acid (7.98 g, 65 mmol), palladium acetate (373 mg, 1.7 mmol) and Dicyclohexyl-[2',4',6'-tris(propan-2-yl)-(1,1'-biphenyl)-2-yl]phosphane (**XPhos**) were suspended in THF (200 mL). To the suspension was added potassium phosphate aqueous solution (2 mol L^{-1}, 76 mL) and the mixture was stirred at reflux temperature for 18.5 hours. After cooling to the room temperature, water and methanol were added to the mixture. The crude product was corrected by filtration, dissolved to toluene and the toluene solution was stirred with activated carbon powder at 100 °C. Then, the clear colorless filtrate was obtained through filtration by using cerite bed. The crude product was purified by recrystallization from toluene to give 2,4-bis(biphenyl-4-yl)-6-(2'-phenyl-1,1':4',1"-terpheny-4"-yl)-1,3,5-triazine (**T2-7668**) as white solid (7.10 g, 10.08 mmol, 63 %).

^{1}H NMR($CDCl_3$): δ= 7.18-7.32 (m, 10H), 7.41(brt, J= 7.5 Hz, 2H), 7.51(brdd, J=7.7, 7.5 Hz, 4H), 7.58 (d, J=7.7 Hz, 1H), 7.73 (brd, J=7.7 Hz, 4H), 7.76-7.81 (m, 2H), 7.83 (d, J=8.3Hz, 4H), 7.90 (d, J=8.3Hz, 2H), 8.87 (d, J=8.3Hz, 4H), 8.88 (d, J=8.3 Hz, 2H) ppm.

^{13}C {^{1}H} NMR($CDCl_3$): δ= 171.4, 171.3, 145.2, 144.5, 141.4, 141.2, 141.0, 140.4, 140.3, 139.5, 135.4, 135.2, 131.3, 129.9, 129.9, 129.6, 129.5, 128.9, 128.0, 128.0, 127.9, 127.4, 127.3, 126.7, 126.7, 126.3 ppm.

MS: m/z = 690 (MH^+), HPLC >99.9%.

Scheme S2 synthesis of **T2-6104**

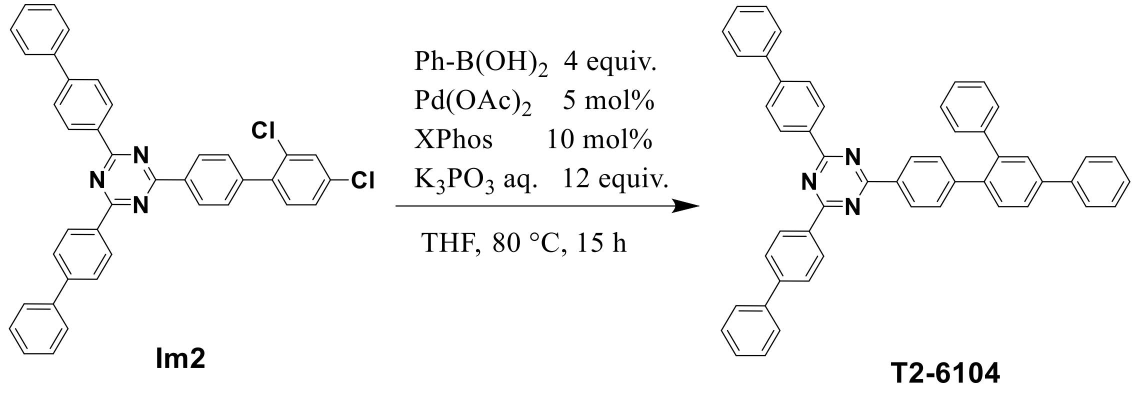

T2-6104. 2,4-Bis(biphenyl-4-yl)-6-(2',4'-dichloro-bephenyl-4-yl)-1,3,5-triazine (**Im2**) was synthesized via known method reported in the previous work.[s1] Under argon atmosphere, **Im2** (4.49 g, 7.4 mmol), phenyl boronic acid (3.63 g, 30 mmol), palladium acetate (87 mg, 0.39 mmol) and XPhos (360 mg, 0.76 mmol) were suspended in THF (150 mL). To the suspension was added potassium phosphate aqueous solution (2 mol L^{-1}, 45 mL) and the mixture was stirred at reflux temperature for 15 hours. After cooling to the room temperature, water and methanol were added to the mixture. After the crude product was corrected by filtration, it was dissolved to toluene and the toluene solution was stirred with activated carbon powder at 100 °C. Then, the clear colorless filtrate was obtained through filtration by using cerite bed. The crude product was purified by recrystallization from toluene to give 2,4-bis(biphenyl-4-yl)-6-(3'-phenyl-1,1':4',1"-terpheny-4"-yl)-1,3,5-triazine (**T2-6104**) as white solid (3.10 g, 4.44 mmol, 60 %).

^{1}H-NMR($CDCl_3$): δ= 7.27-7.29 (m, 5 H), 7.38-7.44 (m, 5H), 7.47-7.52 (m, 6H), 7.61 (dd, J=8.4, 1.1Hz, 1H), 7.71-7.73 (m, 8H), 7.81 (brd, J=8.5Hz, 4H), 8.69 (brd, J=8.4Hz, 2H), 8.84 (brd, J=8.5Hz, 4H) ppm.

^{13}C {^{1}H} NMR($CDCl_3$): 171.4, 171.3, 145.6, 145.2, 141.3, 141.2, 141.0, 140.5, 140.4, 138.9, 135.2, 134.5, 131.1, 130.2, 129.9, 129.6, 129.5, 128.9, 128.8, 128.7, 128.2, 128.0, 127.6, 127.33, 127.30, 127.2, 126.9, 126.3 ppm.

MS: m/z = 690 (M+ H^+), HPLC > 99.9%.

Scheme S3 Synthesis of **T2-6970**

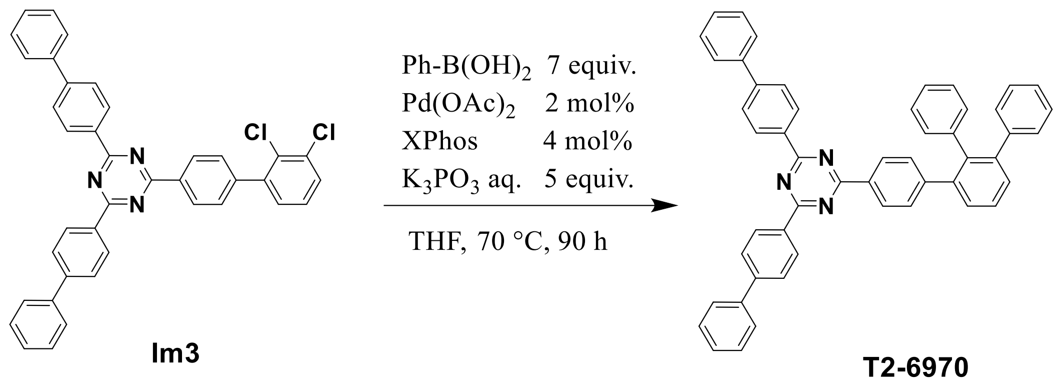

T2-6970. 2,4-Bis(biphenyl-4-yl)-6-(2',3'-dichloro-bephenyl-4-yl)-1,3,5-triazine (**Im3**) was synthesized via known method reported in the previous work.[s2] Under argon atmosphere, **Im3** (9.10 g, 15.1 mmol), phenyl boronic acid (12.8 g, 105 mmol), palladium acetate (68 mg, 0.30 mmol) and XPhos (287 mg, 0.60 mmol) were suspended in THF (300 mL). To the suspension was added potassium phosphate aqueous solution (2 mol L^{-1}, 38 mL) and the mixture was stirred at reflux temperature for 90 hours. After cooling to the room temperature, water and methanol were added to the mixture. After the crude product was corrected by filtration, it was dissolved to toluene and the toluene solution was stirred with activated carbon powder at 100 °C. Then, the clear colorless filtrate was obtained through filtration by using cerite bed. The crude product was purified by recrystallization from toluene to give 2,4-bis(biphenyl-4-yl)-6-(2'-phenyl-1,1':3',1"-terpheny-4"-yl)-1,3,5-triazine (**T2-6970**) as white solid (6.9 g, 10.0 mmol, 66 %).

^{1}H NMR($CDCl_3$): δ= 6.89-6.94 (m, 2 H), 6.98-7.05 (m, 3H), 7.08-7.13 (m, 2H), 7.17 (m, 3H), 7.32 (dt, J=8.4, 1.7Hz, 2H), 7.42 (dd, J=8.4, 8.1Hz, 2H), 7.47-7.57 (m, 7H), 7.71 (d, 4H), 7.80 (brd, J=8.5Hz, 4H) 8.62 (brd, J=8.3Hz, 2H), 8.83 (brd, J=8.5Hz, 4H) ppm.

^{13}C {^{1}H} NMR($CDCl_3$): δ= 171.4, 171.3, 146.6, 145.2, 142.2, 141.8, 141.3, 140.4, 139.3, 139.2, 135.2, 134.0, 131.6, 130.3, 130.1, 129.9, 129.52, 129.46, 128.9, 128.3, 128.0, 127.6, 127.5, 127.4, 127.32, 127.29, 126.3, 126.2 ppm.

MS: m/z = 690 (M+ H^+), HPLC > 99.9%.

Scheme S4 Synthesis of **T2-7191**

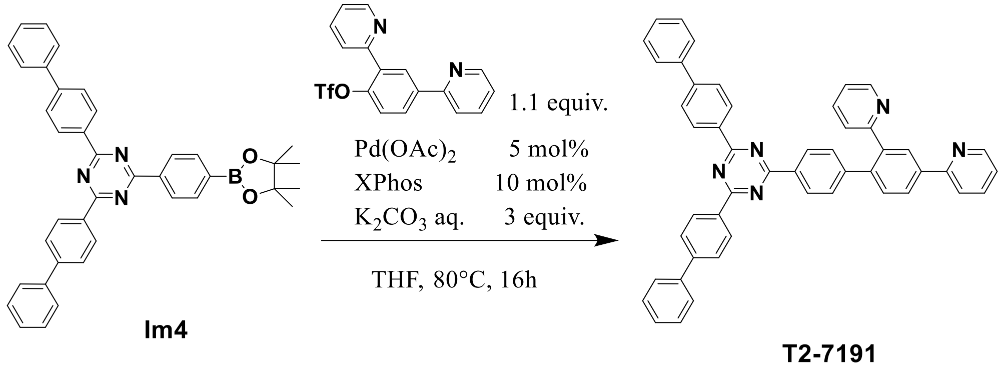

T2-7191. 2,4-Bis(biphenyl-4-yl)-6-[(4,4,5,5-tetrametyl-1,3,2-dioxaborolan-2-yl)-phenyl]-1,3,5-triazine (**Im4**) was synthesized via known method reported in the previous work.[s3] Under argon atmosphere, **Im4** (1.33 g, 3.18 mmol), 2,4-bis(pyridine-2-yl)-phenyl trifluoromethanesulfonate (1.87 g, 3.50 mmol), palladium acetate (36 mg, 0.16 mmol) and XPhos (152 mg, 0.32 mmol) were suspended in THF (32 mL). To the suspension was added potassium phosphate aqueous solution (2 mol L^{-1}, 4.8 mL) and the mixture was stirred at reflux temperature for 16 hours. After cooling to the room temperature, water and methanol were added to the mixture. After the crude product was corrected by filtration, it was dissolved to toluene and the toluene solution was stirred with activated carbon powder at 100 °C. Then, the clear colorless filtrate was obtained through filtration by using cerite bed. The crude product was purified by recrystallization from toluene to give 2,4-bis(biphenyl-4-yl)-6-[2,4-(pyridine-2-yl)-biphenyl-4'-yl]-1,3,5-triazine (**T2-7191**) as white solid (1.25 g, 1.81 mmol, 57 %).

^{1}H NMR($CDCl_3$): δ= 7.07 (d, J=7.9Hz, 1 H), 7.17(m, 1H), 7.39-7.54 (m, 10H), 7.68 (d, J=8.0Hz, 1H), 7.72 (d, J=7.4Hz, 4H), 7.76-7.84 (m, 5H), 7.90 (d, J=8.0 Hz, 1H), 8.22 (dd, J=8.0, 1.9Hz, 1H), 8.36 (d, J=1.8Hz, 1H), 8.68-8.76 (m, 4H), 8.85 (d, J=8.4Hz, 4H) ppm.

^{13}C {^{1}H} NMR($CDCl_3$): δ= 171.33, 171.29, 159.0, 156.6, 149.8, 149.6, 145.4, 145.2, 140.5, 140.4, 140.1, 139.2, 136.8, 135.6, 135.1, 134.8, 131.0, 130.0, 129.5, 129.3, 128.9, 128.8, 128.0, 127.54, 127.49, 127.30, 127.27, 127.0, 125.4, 122.3, 121.7, 120.7 ppm.

MS: m/z= 692 (M+ H^{+}), HPLC > 99.9%.

Scheme S5 Synthesis of **T4-442**

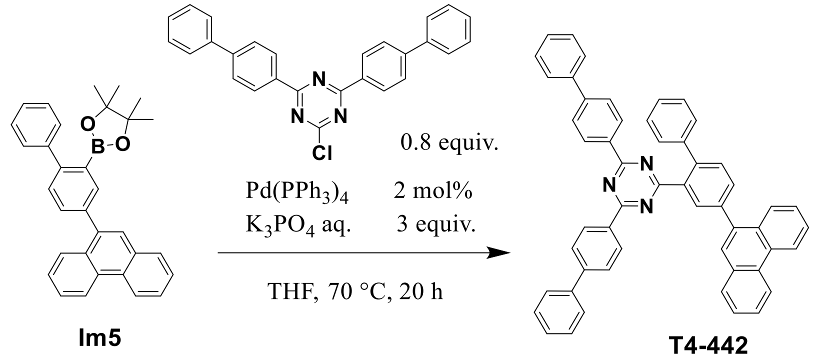

T4-442. [4-(phenathlene-9-yl)-1,1'-biphenyl-2-yl]-4,4,5,5-tetrametyl-1,3,2-dioxaborolan (**Im5**) was synthesized via known method reported in the previous work.[s2] Under argon atmosphere, **Im5** (3.0 g, 6.6 mmol), 2-chloro-4,6-bis(biphenyl-4-yl)-1,3,5-trizine (2.3 g, 5.5 mmol), tetrakis(triphenylphosphine) palladium ($Pd(PPh_3)_4$, 127 mg, 0.11 mmol) were suspended in THF (110 mL). To the suspension was added potassium phosphate aqueous solution (2 mol L^{-1}, 8 mL) and the mixture was stirred at reflux temperature for 20 hours. After cooling to the room temperature, water and methanol were added to the mixture. After the crude product was corrected by filtration, it was dissolved to toluene and the toluene solution was stirred with activated carbon powder at 100 °C. Then, the clear colorless filtrate was obtained through filtration by using cerite bed. The crude product was purified by recrystallization from toluene/1-butylalcohol to give 2,4-bis(biphenyl-4-yl)-6-[4-(phenanthrene-9-yl)-(1,1'-biphenyl)-2-yl]-1,3,5-triazine (**T4-442**) as white solid (1.5 g, 2.1 mmol, 38 %).

^{1}H NMR($CDCl_3$): δ= 7.31-7.50 (m, 11H), 7.58-7.73 (m, 13H), 7.81 (dd, J=7.9,1.9Hz, 1H), 7.87 (s, 1H), 7.98 (dd, J=7.9, 1.4Hz, 1H), 8.10 (dd, J=8.7,1.3Hz, 1H), 8.40 (brd, J=8.4Hz, 4H), 8.56 (d, J=1.8Hz, 1H), 8.78 (d, J=8.5Hz, 1H), 8.84 (d, J=8.5Hz, 1H) ppm.

^{13}C {^{1}H} NMR($CDCl_3$): δ= 174.2, 171.0, 145.1, 142.5, 142.4, 140.3, 140.2, 137.9, 136.1, 134.8, 133.0, 132.5, 131.5, 131.1, 130.7, 130.1, 129.4, 129.2, 128.9, 128.8, 128.3, 127.94, 127.87, 127.2, 127.1, 126.9, 126.79, 126.78, 126.72, 126.6, 123.0, 122.6 ppm.

MS: m/z= 714 (M+ H^+), HPLC > 99.9%.

Scheme S6 Synthesis of **T4-2766**

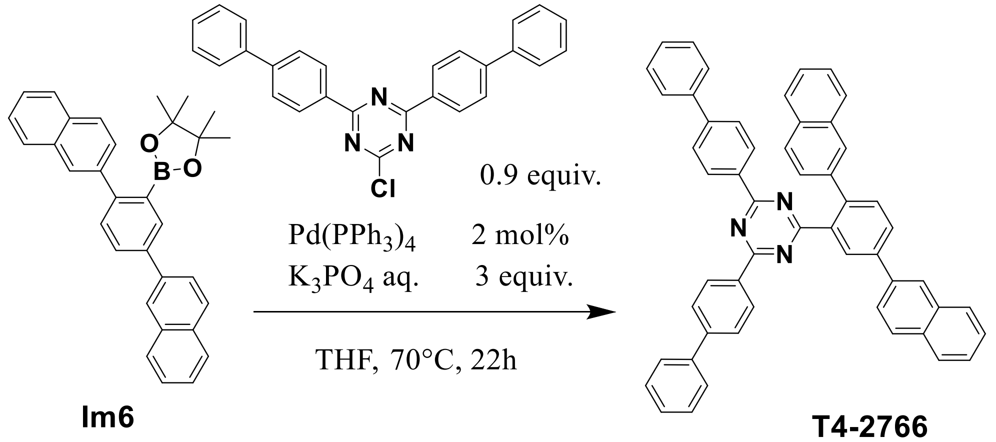

T4-2766. [2,5-(naphthalene-2-yl)-phenyl]-4,4,5,5-tetrametyl-1,3,2-dioxaborolan (**Im6**) was synthesized via known method reported in the previous work.[s2] Under argon atmosphere, **Im6** (4.8 g, 10.5 mmol), 2-chloro-4,6-bis(biphenyl-4-yl)-1,3,5-trizine (4.5 g, 9.5 mmol), $Pd(PPh_3)_4$ (220 mg, 0.19 mmol) were suspended in THF (190 mL). To the suspension was added potassium phosphate aqueous solution (2 mol L^{-1}, 14 mL) and the mixture was stirred at reflux temperature for 22 hours. After cooling to the room temperature, water and methanol were added to the mixture. The precipitated crude was corrected by filtration and washed water and acetone. Crude product was dissolved to toluene and the toluene solution was stirred with activated carbon powder at 100 °C. Then, the clear colorless filtrate was obtained through filtration by using cerite bed. The crude product was purified by recrystallization from the filtrate to give 2,4-bis(biphenyl-4-yl)-6-[2,5-(naphtharene-2-yl)-phenyl]-1,3,5-triazine (**T4-2766**) as white solid (1.5 g, 2.1 mmol, 38 %).

^{1}H NMR($CDCl_3$): δ= 7.39 (m, 3H), 7.44-7.65 (m, 16H), 7.74 (d, J=8.3Hz, 1H), 7.78 (d, J=8.1Hz, 1H), 7.83 (brd, J=8.5Hz, 1H), 7.90-8.10 (m, 7H), 8.26 (m, 5H), 8.85 (d, J=1.8Hz, 1H) ppm.

^{13}C {^{1}H} NMR($CDCl_3$): δ= 172.8, 170.0, 144.0, 141.4, 139.7, 139.4, 139.3, 136.7, 135.4, 133.7, 132.8, 132.7, 131.8, 131.4, 129.4, 128.9, 128.4, 127.8, 127.7, 127.3, 127.0, 126.9, 126.7, 126.5, 126.2, 126.1, 126.0, 125.5, 125.2, 125.1, 124.8, 124.5.

MS: m/z= 714 (M+ H^+), HPLC purity >99.5%.

Scheme S7 Synthesis of **T2-3317**

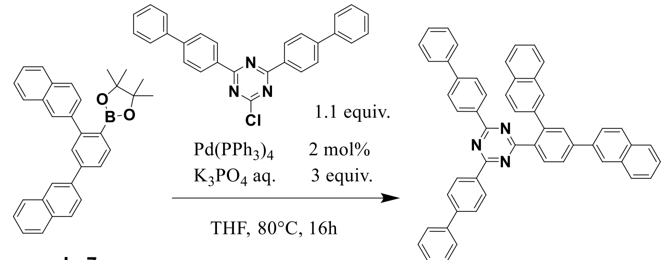

T2-3317. [2,4-(naphthalene-2-yl)-phenyl]-4,4,5,5-tetrametyl-1,3,2-dioxaborolan (**Im7**) was synthesized via known method reported in the previous work.[s4] Under argon atmosphere, **Im7** (3.0 g, 6.6 mmol), 2-chloro-4,6-bis(biphenyl-4-yl)-1,3,5-trizine (2.5 g, 6.0 mmol), $Pd(PPh_3)_4$ (238 mg, 0.12 mmol) were suspended in THF (200 mL). To the suspension was added potassium phosphate aqueous solution (2 mol L^{-1}, 9 mL) and the mixture was stirred at reflux temperature for 22 hours. After cooling to the room temperature, the resulting mixture was diluted with water, extracted with toluene. The combined organic layers were dried over Na_2SO_4 and filtered. To the concentrated filtrate was added ethanol and the precipitated solid was corrected by filtration. After washing the precipitated solid by water and ethanol, the crude product was dissolved to toluene and the toluene solution was stirred with activated carbon powder at 100 °C. Then, the clear colorless filtrate was obtained through filtration by using cerite bed. The crude product was purified by recrystallization from the filtrate to give 2,4-bis(biphenyl-4-yl)-6-[2,4-(naphthalene-2-yl)-phenyl]-1,3,5-triazine (**T2-3317**) as white solid (1.8 g, 2.5 mmol, 27%).

^{1}H NMR($CDCl_3$): δ= 7.39 (dt, J=7.7,0.7Hz, 2H), 7.43 (dd, J=7.9,1.7Hz, 1H), 7.45-7.58(m, 12H), 7.61 (m, 4H), 7.78 (d, J=7.9Hz, 1H), 7.85 (brd, J=8.2Hz, 1H), 7.91 (m, 2H), 7.93-8.04 (m, 5H), 8.11(brs, 1H), 8.24 (m, 5H), 8.71(d, J=7.9Hz, 1H) ppm.

^{13}C {^{1}H} NMR($CDCl_3$): δ= 173.3, 170.9, 145.0, 144.2, 143.8, 141.1, 140.4, 137.4, 134.8, 134.6, 133.9, 133.7, 133.0, 132.5, 132.3, 130.9, 129.4, 128.9, 128.7, 128.4, 128.0, 127.9, 127.74, 127.71, 127.5, 127.2, 127.0, 126.6, 126.5, 126.4, 126.3, 126.2, 125.8, 125.4 ppm.

MS: $[M]^+$ = 714 (M+ H^+), HPLC purity >99.9%.

Scheme S8 Synthesis of **T1-4799**

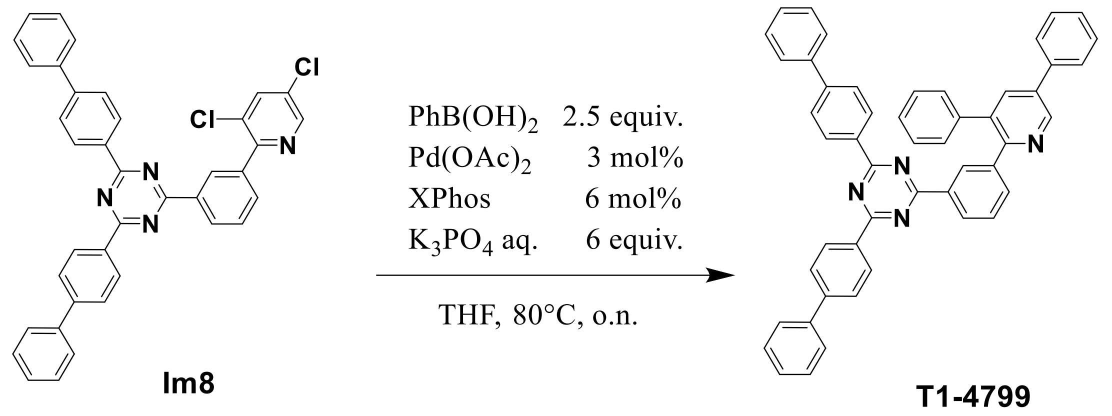

T1-4799. 2,4-bis(biphenyl-4-yl)-6-[3-(3,5-dichloropyridine-2-yl)-phenyl]-1,3,5-triazine (**Im8**) was synthesized via known method reported in the previous work.[s5] Under argon atmosphere, **Im8** (7.28 g, 12.0 mmol), phenyl boronic acid (3.66 g, 30.0 mmol), and palladium acetate (84 mg, 0.36 mmol) and XPhos (348 mg, 0.72 mmol) were suspended in THF (120 mL). To the suspension was added potassium phosphate aqueous solution (4 mol L^{-1}, 18 mL) and the mixture was stirred at reflux temperature for overnight. After cooling to the room temperature, to the resulting mixture was added water and methanol. The precipitated solid was filtrated and washed by water and methanol. The crude product was dissolved to $CHCl_3$ and the solution was stirred with activated carbon powder at room temperature. Then, the colorless filtrate was obtained through filtration by using cerite bed. The crude product was purified by recrystallization from the filtrate to give 2,4-bis(biphenyl-4-yl)-6-[3-(3,5-diphenylpyridine-2-yl)-phenyl]-1,3,5-triazine (**T1-4799**) as white solid (5.81 g, 8.41 mmol, 70%).

^{1}H NMR($CDCl_3$): δ= 7.26-7.54 (m, 15H), 7.70-7.74 (m, 7H), 7.79 (d, J=8.5Hz, 1H), 8.00 (d, J=2.3Hz, 1H), 8.72 (dd, J=7.6, 1.1Hz, 1H), 8.78 (d, J=8.4Hz, 4H), 8.84 (s, 1H) ppm.

^{13}C {^{1}H} NMR($CDCl_3$): δ= 171.3, 171.2, 155.3, 146.9, 145.1, 140.4, 140.3, 139.8, 137.4, 137.1, 136.2, 135.9, 135.3, 135.1, 134.0, 130.8, 129.7, 129.5, 129.2, 128.9, 128.6, 128.5, 128.4, 128.2, 128.0, 127.6, 127.5, 127.3, 127.24, 127.17, 126.9 ppm.

MS: m/z = 691 (M+ H^+), HPLC purity > 99.9%.

Scheme S9 Synthesis of **T1-5248**

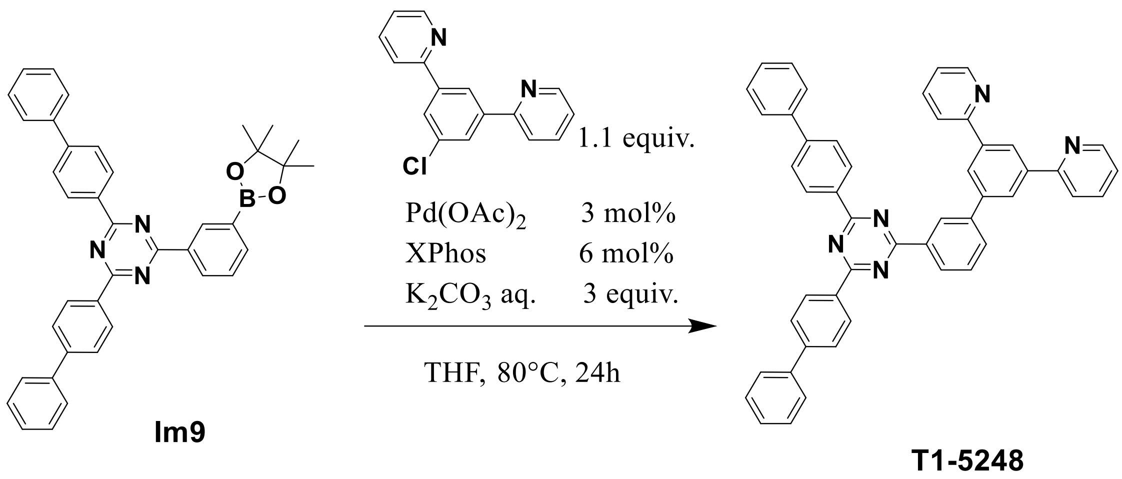

T1-5248. 2,4-Bis(biphenyl-4-yl)-6-[3-(4,4,5,5-tetrametyl-1,3,2-dioxaborolan-2-yl)-phenyl]-1,3,5-triazine (**Im9**) was synthesized via known method reported in the previous work.[s6] Under argon atmosphere, **Im9** (4.89 g, 8.28 mmol), 5-chloro-1,3-di(pyridine-2-iyl)-benzene (2.52 g, 9.31 mmol), and palladium acetate (60 mg, 0.29 mmol) and XPhos (254 mg, 0.58 mmol) were suspended in THF (80 mL). To the suspension was added potassium carbonate aqueous solution (2 mol L^{-1}, 13 mL) and the mixture was stirred at reflux temperature for 24 h. After cooling to the room temperature, to the resulting mixture was added water and methanol. The precipitated solid was filtrated and washed by water and methanol. The crude product was dissolved to $CHCl_3$ and the solution was stirred with activated carbon powder at room temperature. Then, the clear colorless filtrate was obtained through filtration by using cerite bed. The crude product was purified by recrystallization from toluene to give 2,4-bis(biphenyl-4-yl)-6-[3',5'-di(pyridine-2-yl)-biphenyl-3-yl]-1,3,5-triazine (**T1-5248**) as white solid (5.00 g, 7.17 mmol, 87%).

^{1}H-NMR($CDCl_3$): δ= 7.26-7.54 (m, 15H), 7.70-7.74 (m, 7H), 7.79 (d, J=8.5Hz, 1H), 8.00 (d, J=2.3Hz, 1H), 8.72 (dd, J=7.6, 1.1Hz, 1H), 8.78 (d, J=8.4Hz, 4H), 8.84 (s, 1H) ppm.

^{13}C {^{1}H} -NMR ($CDCl_3$): δ= 171.7, 171.5, 157.2, 149.8, 145.2, 142.2, 141.7, 140.6, 140.4, 136.9, 136.8, 135.2, 131.8, 129.6, 129.1, 128.9, 128.3, 128.0, 127.9, 127.4, 127.3, 126.7, 124.9, 122.5, 121.0 ppm.

MS: $[MH]^+$ = 692, HPLC purity > 99.9%.

Histograms of training datasets (Figure S2)

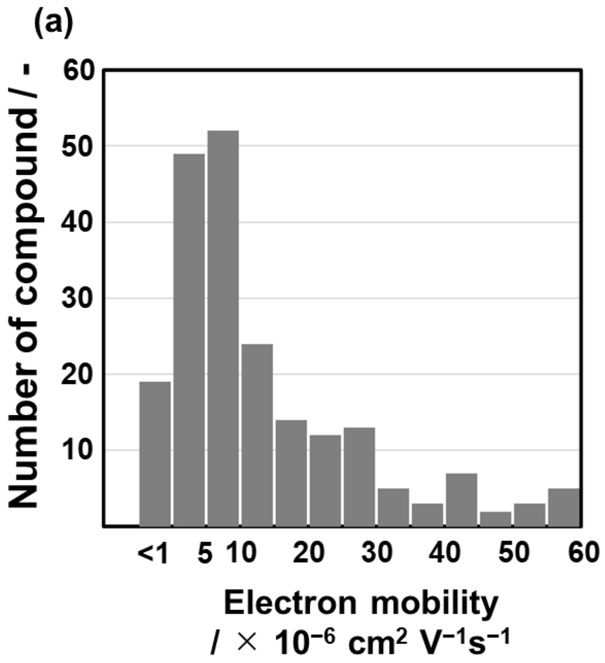

Figure S2 Data distribution of the initial datasets. (a) electron mobility estimated from the EOD (b) glass transition temperature.

Initial screening of machine learning models on PyCaret (Figure S3)

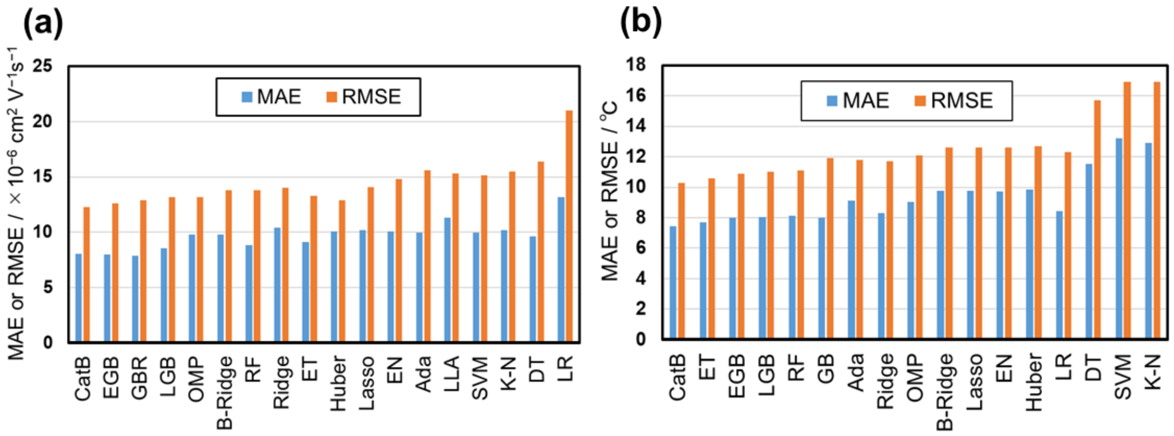

Figure S3 Comparison of training algorithms based on 2D descriptors before hyperparameter tuning for (a) μ_e prediction, (b) T_g prediction. Abbreviations in the horizontal axis: CatB for catboost regressor; EGB for extreme gradient boosting; GBR for gradient boosting regressor; LGB for light boosting regressor; OMP for orthogonal matching pursuit; B-Ridge for bayesian ridge; RF for random forest; ET for extra tree; EN for elastic net; Ada for Adaboost regressor; LLA for lasso least angle regression; SVM for support vector machine; K-N for K neighbors regressor; DT for decision tree; LR for linear regressor.

Whole processes were performed by using Pandas 1.1.2 and PyCaret 2.1.2 environment. The model comparison was performed by "compare_model()" function of PyCaret. After train/test separation, various types of prediction models were trained with default values of hyperparameters. The MAE and RMSE score were calculated from blind test data. After that, we selected the algorithms showing superior prediction scores. The hyperparameter tuning was performed by 5-fold cross validation scheme based on the MAE score by using "tune_model()" function of PyCaret. The random grid search algorithm was used for the hyperparameter optimization. Although the boosting-tree type models showed higher scores initially, they resulted in overfitting of training data. In the tuning process of the boosting-tree type algorithms, MAE of training dataset became close to zero, but MAE of test data did not decrease through the further optimization. The behavior seems to be derived from the small amount of the training dataset.

Comparison of T_g prediction models (Table S1)

Table S1 Properties of the previously reported T_g prediction models.

Algorithm	Compounds	Data *N*	MAE / K	R^2	Reference
Linear regression	OLED-related + typical solvents	103	13.9	0.976	[s7]
Linear regression	OLED-related + hydrocarbons	80	n.a.	0.927	[s8]
Linear regression	OLED-related	80	7.7	0.930	[s9]
SVM	OLED-related	66	5.5	0.963	[s10]
LightGBM	Any compounds	1944	17.1	0.77	[s11]
	OLED-related†	40†	8.9†	0.90†	[s11]
KPLS	OLED-related	250	n.a.	0.86	[s12]
Extra tree with 2D descriptors	Triazines	554	5.9‡	0.863‡	This work
Extra tree with 3D descriptors	Triazines	554	3.4	0.942	This work

† The prediction model based on 1944 literature data was applied to 40 OLED-related compounds.

‡ The T_g(2D) and T_g(3D) models were trained on the same train/test separation. The MAE and R^2 values are slightly different from Table 1 due to the change of the train/test separation.

Examples of the structure-predicted properties in the last screening step (Table S2)

Table S2. The triazine compounds excluded in the last screening step: molecular structures, its calculated LUMO levels, predicted T_g(2D), T_g(3D), μ_e, and the reason not to be chosen as the potentially practical ETL materials.

Structure	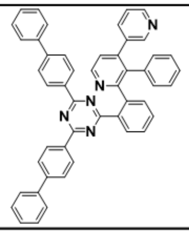	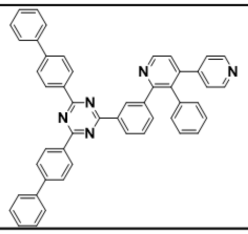	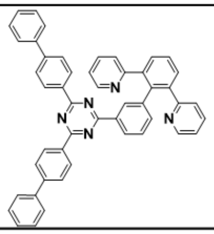	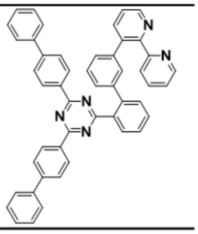
ID	T3-7992	T1-6906	T1-7956	T3-8866
LUMO level / eV	−1.89	−1.88	−1.81	−1.86
Predicted μ_e $\times 10^{-6}$/ $cm^2\ V^{-1}s^{-1}$	15.5	19.7	15.8	10.8
Predicted T_g(2D) / °C	122	123	121	121
Predicted T_g(3D) / °C	123	126	124	120
Reason for exclusion	unavailability of the intermediate compounds	unavailability of the intermediate compounds	need for tin reagent	relatively low properties

Structure	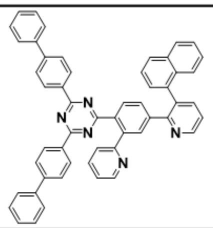	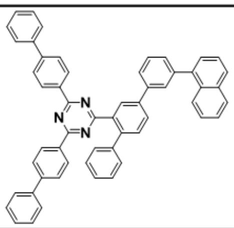	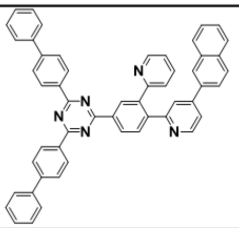	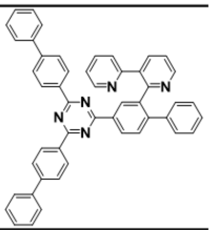
ID	T3-3289	T4-241	T2-2944	T2-2816
LUMO level / eV	−1.85	−1.87	−1.88	−1.85
Predicted μ_e $\times 10^{-6}$/ $cm^2\ V^{-1}s^{-1}$	13.8	13.1	11.4	13.6
Predicted T_g(2D) / °C	130	124	124	129
Predicted T_g(3D) / °C	129	129	130	126
Reason for exclusion	need for tin reagent	similarity to T4-442	need for many synthesis steps	need for tin reagent

Luminescence spectra and other properties of the fabricated OLED devices (Figure S4)

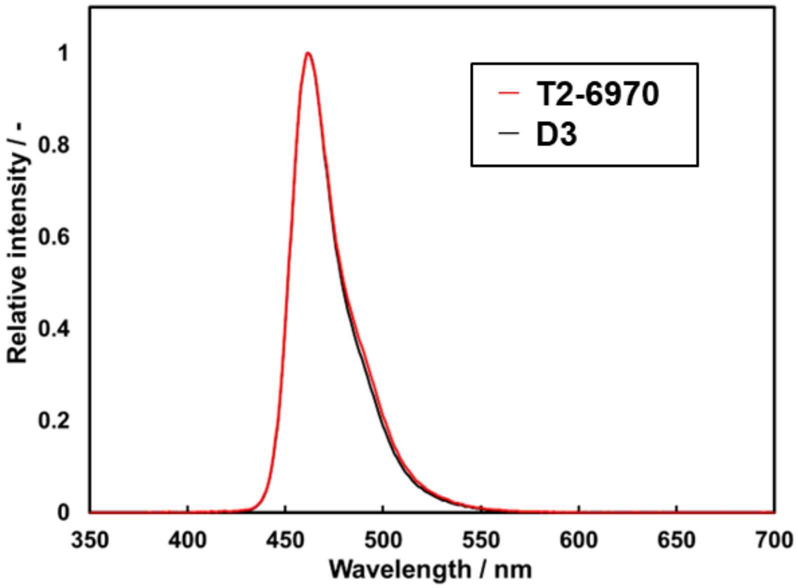

Figure S4. Emission spectra of the OLED device fabricated in this work.

The slight difference in the range from 470 nm to 500 nm seems to be derived from the change of carrier balance.

Table S3 Properties of the fabricated OLED devices at 10 mA cm^{-2} of current density.

ETL	CIEx	CIEy	EQE /%	Terminal voltage / V	Emission intensity / cd m^{-2}	Current efficiency / cd A^{-1}	Power efficiency / cd W^{-1}
**T2-6970**	0.128	0.097	6.29	3.53	506	5.06	4.50
**D3**	0.129	0.092	6.31	3.74	488	4.88	4.10

Raw NMR and LC-MS data of the synthesized compounds

(a)

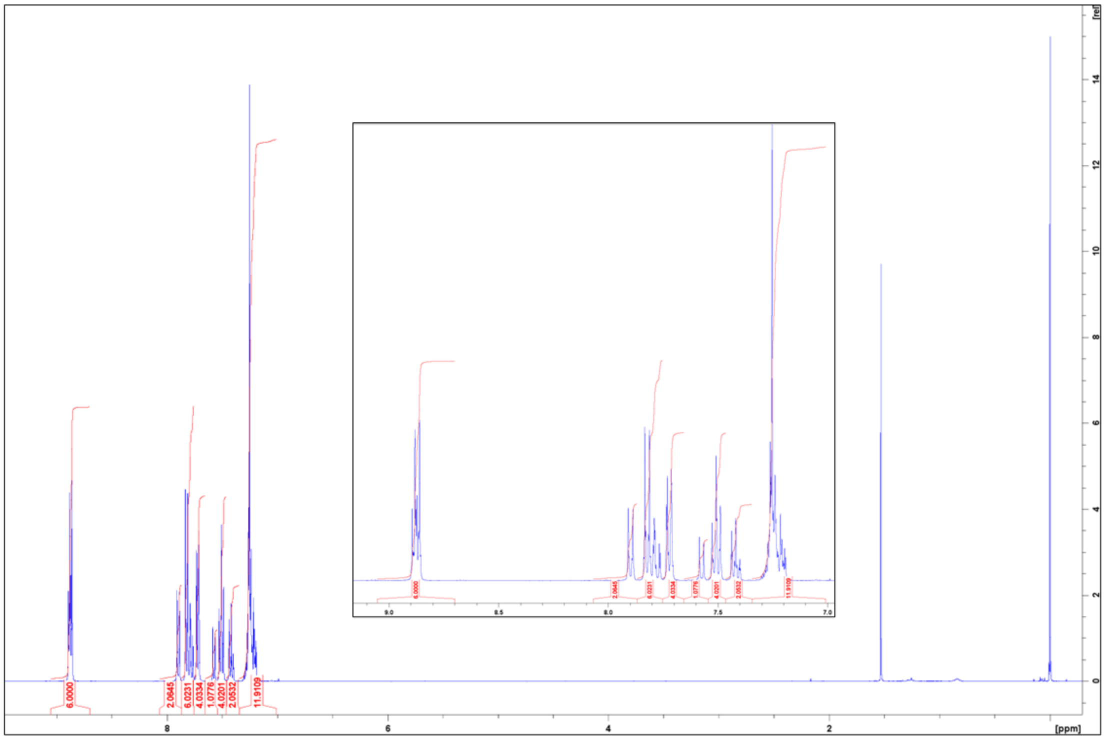

(b)

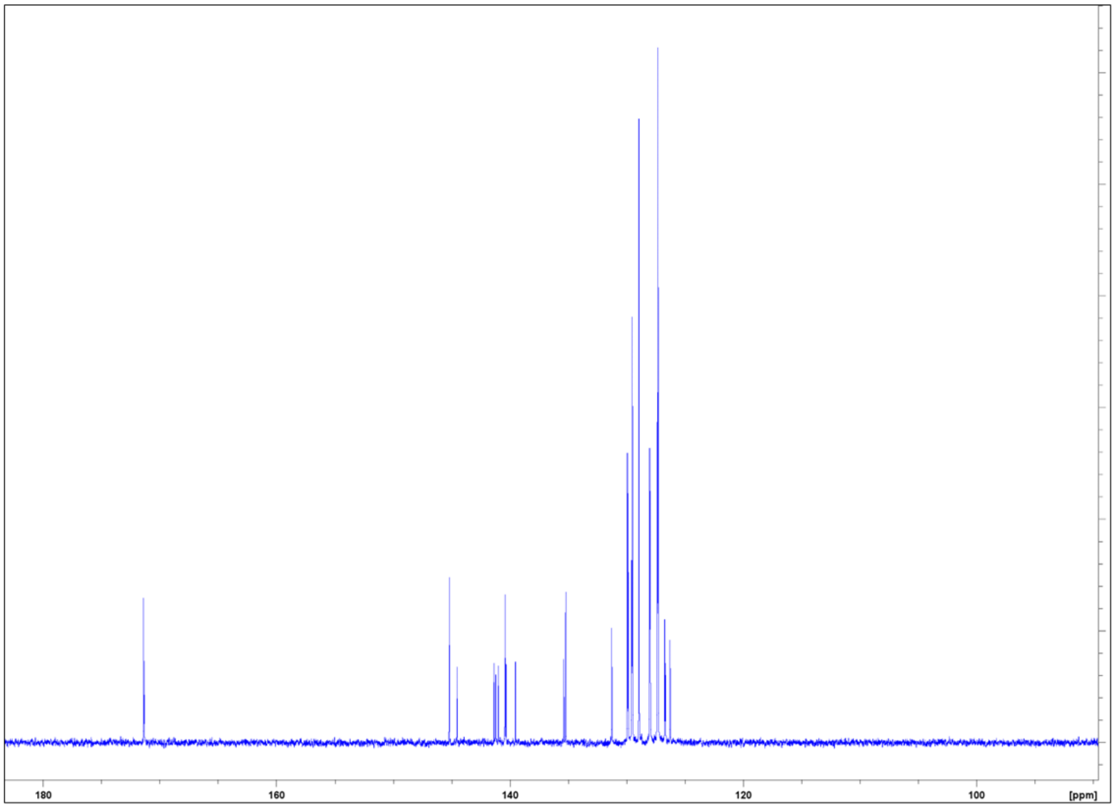

(c)

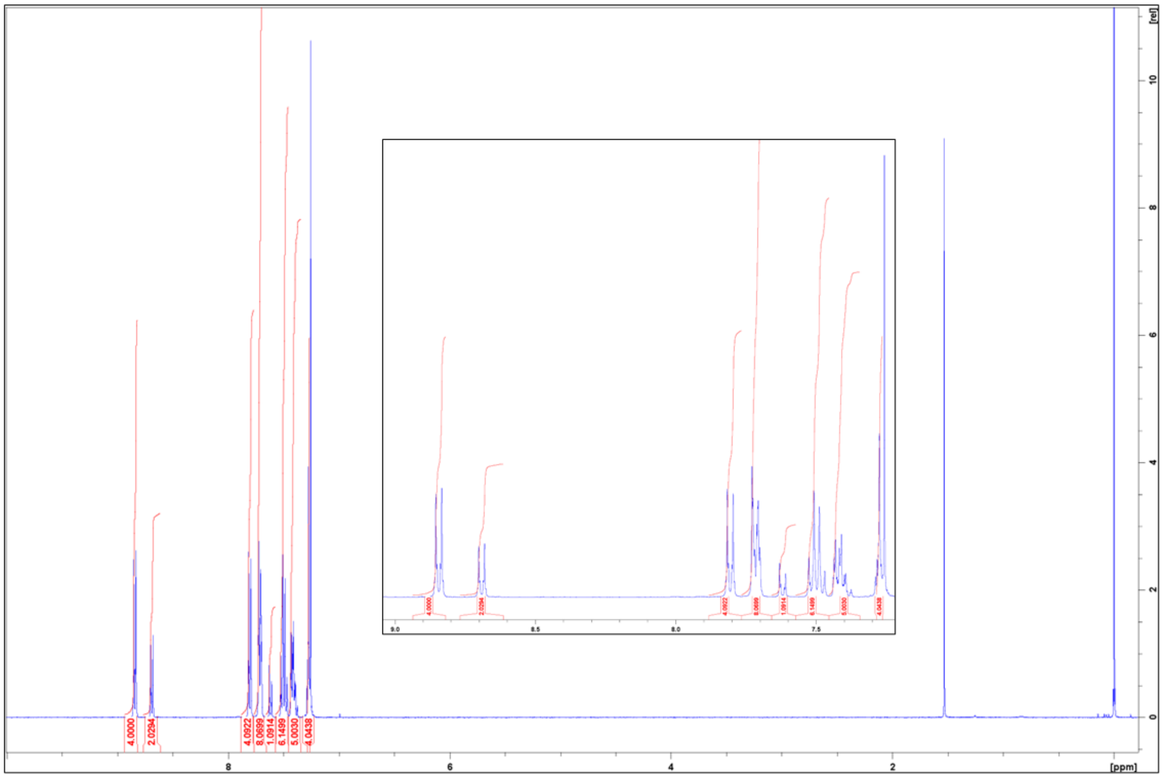
4.0000
2.0294
4.0922
8.0699
1.0914
6.1499
5.0030
4.0438
8
6
4
2
[ppm]
[rel]
10
8
6
4
2
0
9.0
8.5
8.0
7.5

(d)

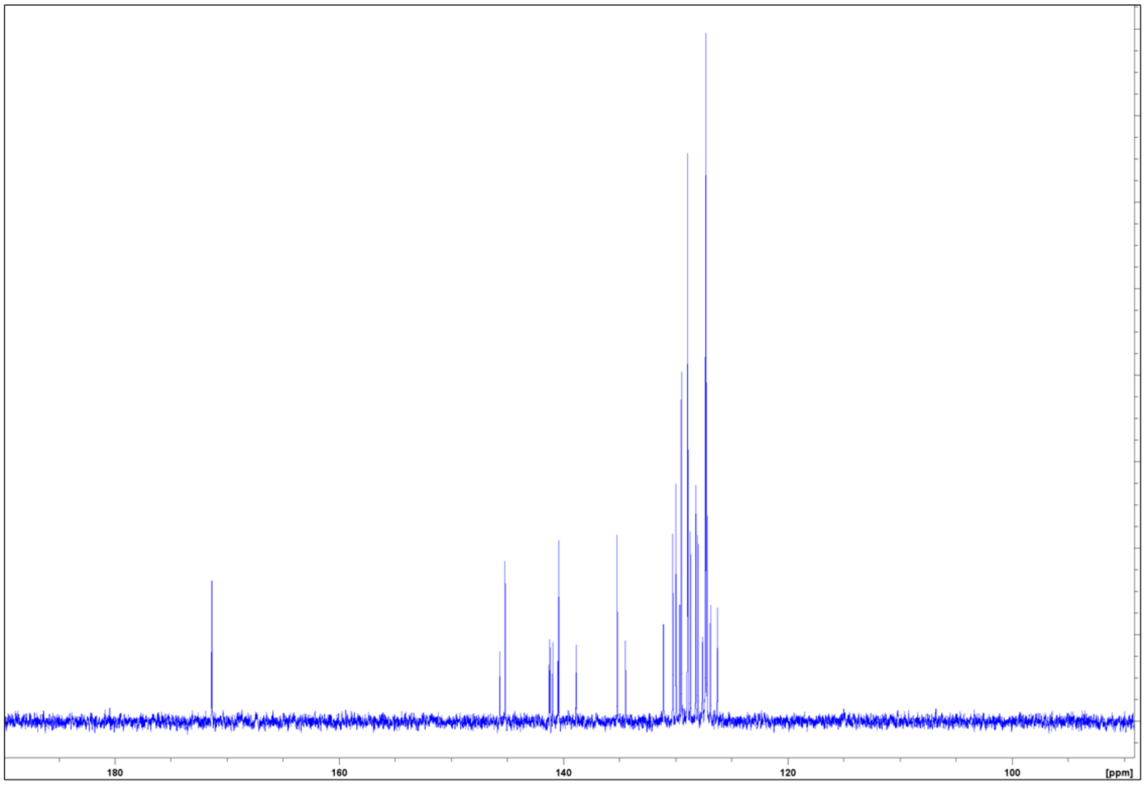
180
160
140
120
100
[ppm]

(e)

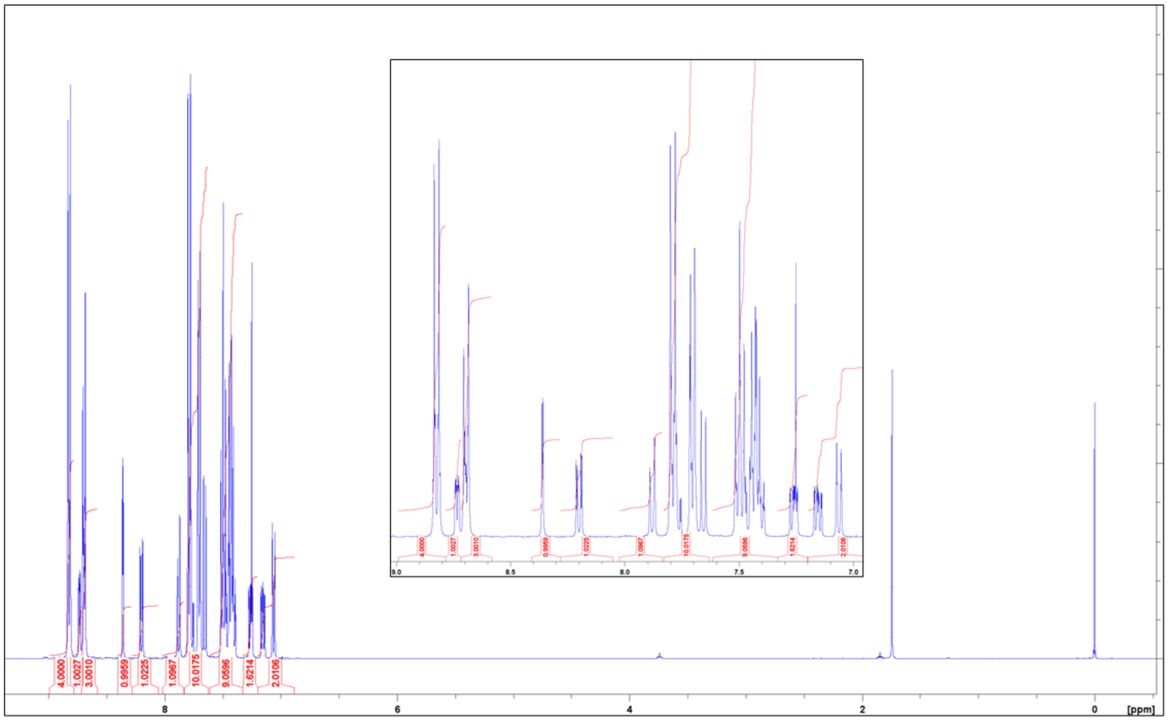
4.0000
1.0027
3.0010
0.9959
1.0225
1.0967
10.0175
9.0596
1.6214
2.0106
8
6
4
2
0
[ppm]
9.0
8.5
8.0
7.5
7.0

(f)

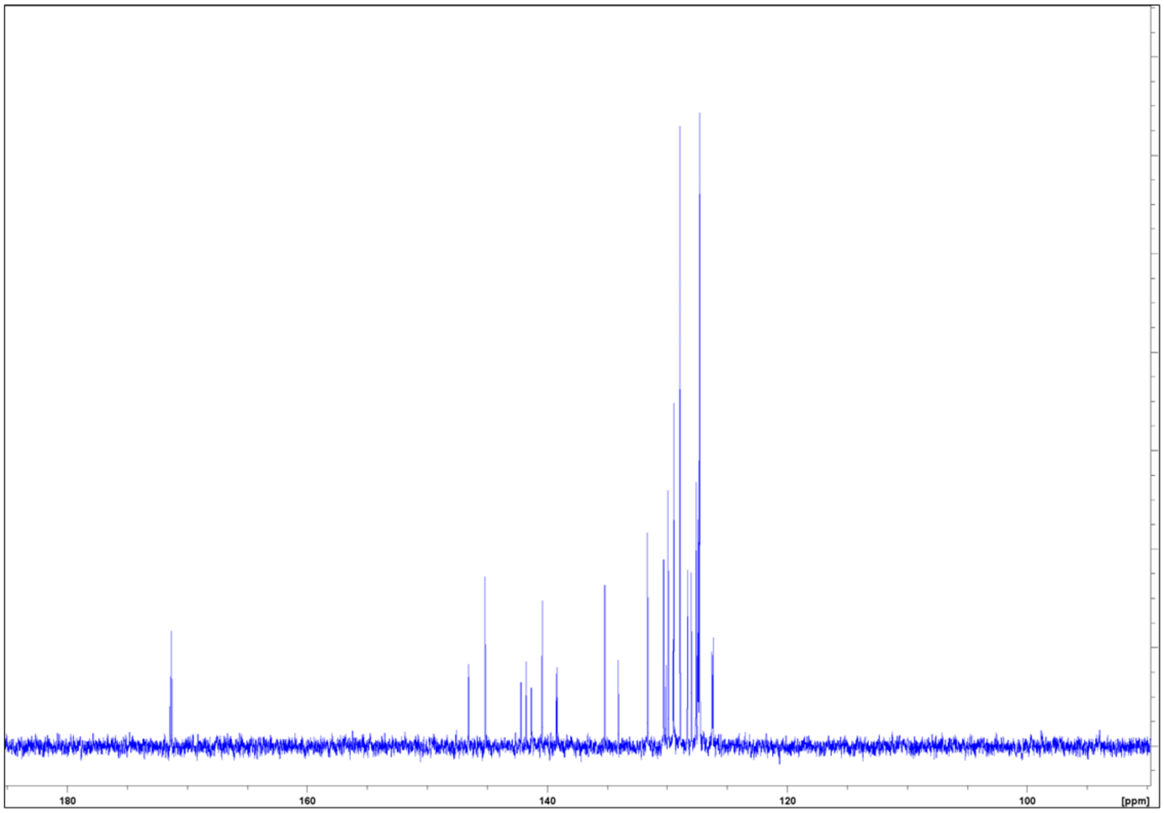
180
160
140
120
100
[ppm]

(g)

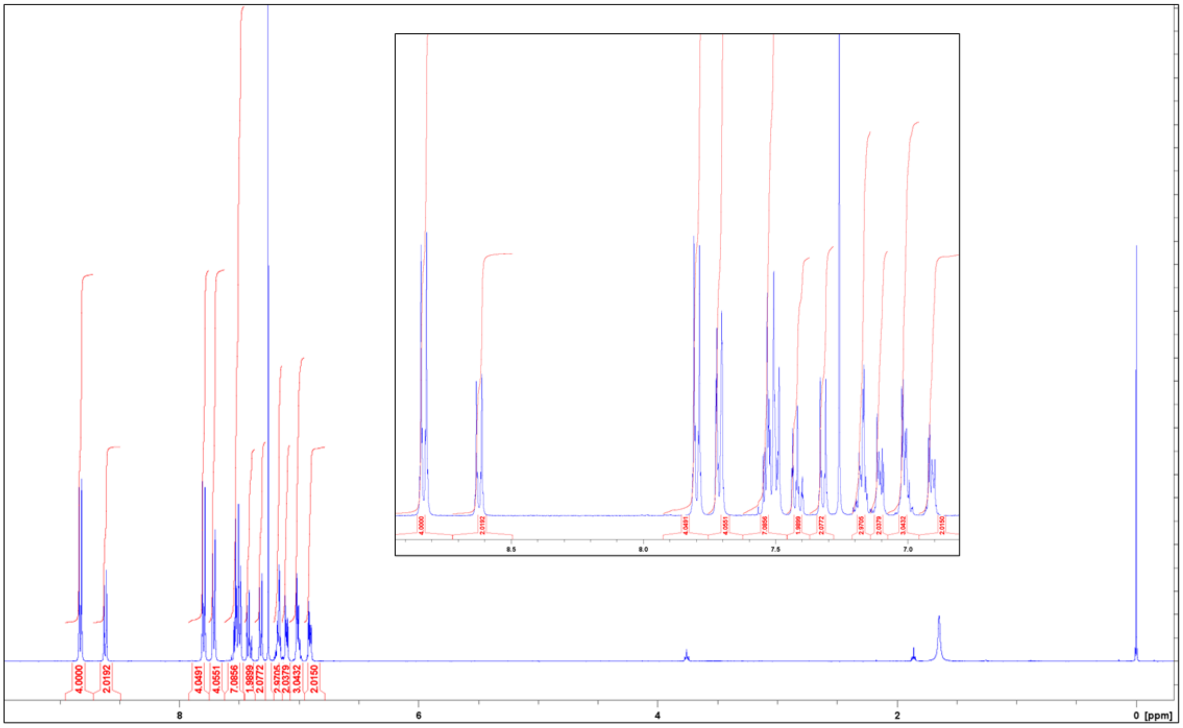
4.0000
2.0192
4.0491
4.0551
7.0856
1.9899
2.0772
2.9705
2.0379
3.0432
2.0150
8
6
4
2
0 [ppm]

(h)

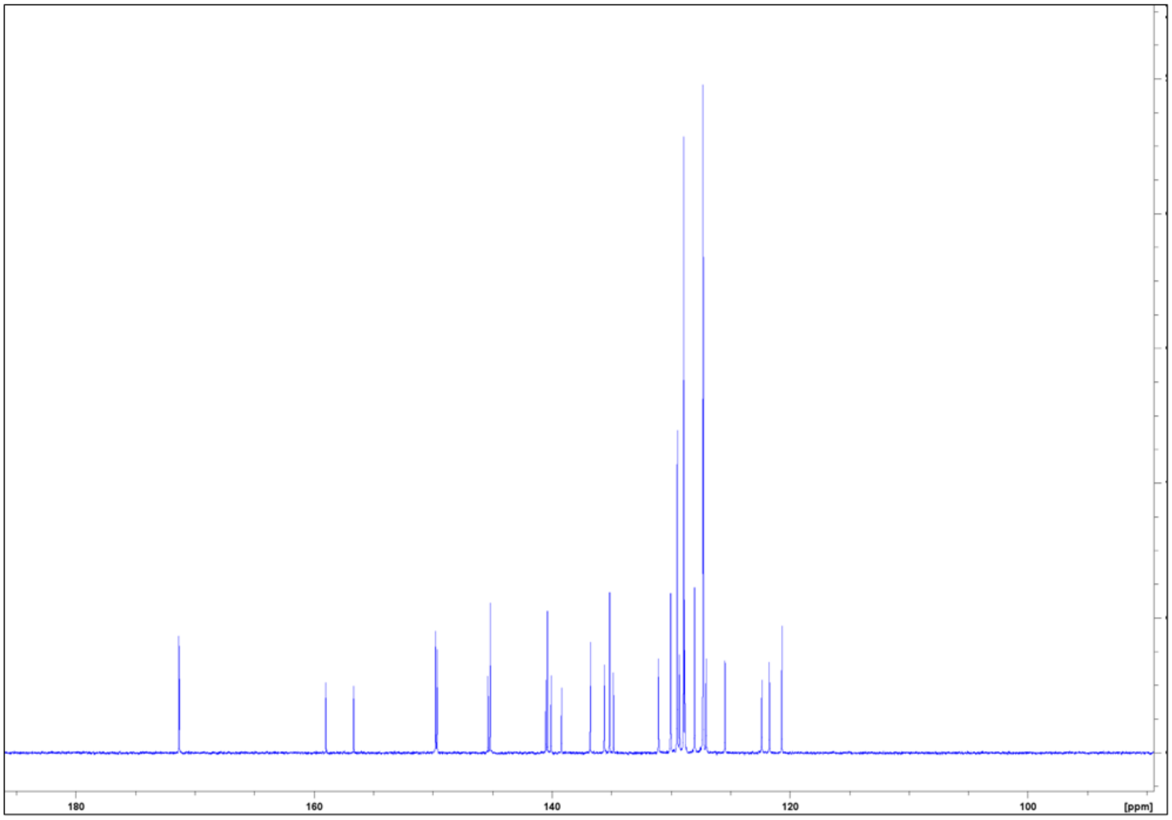
180
160
140
120
100
[ppm]

(i)

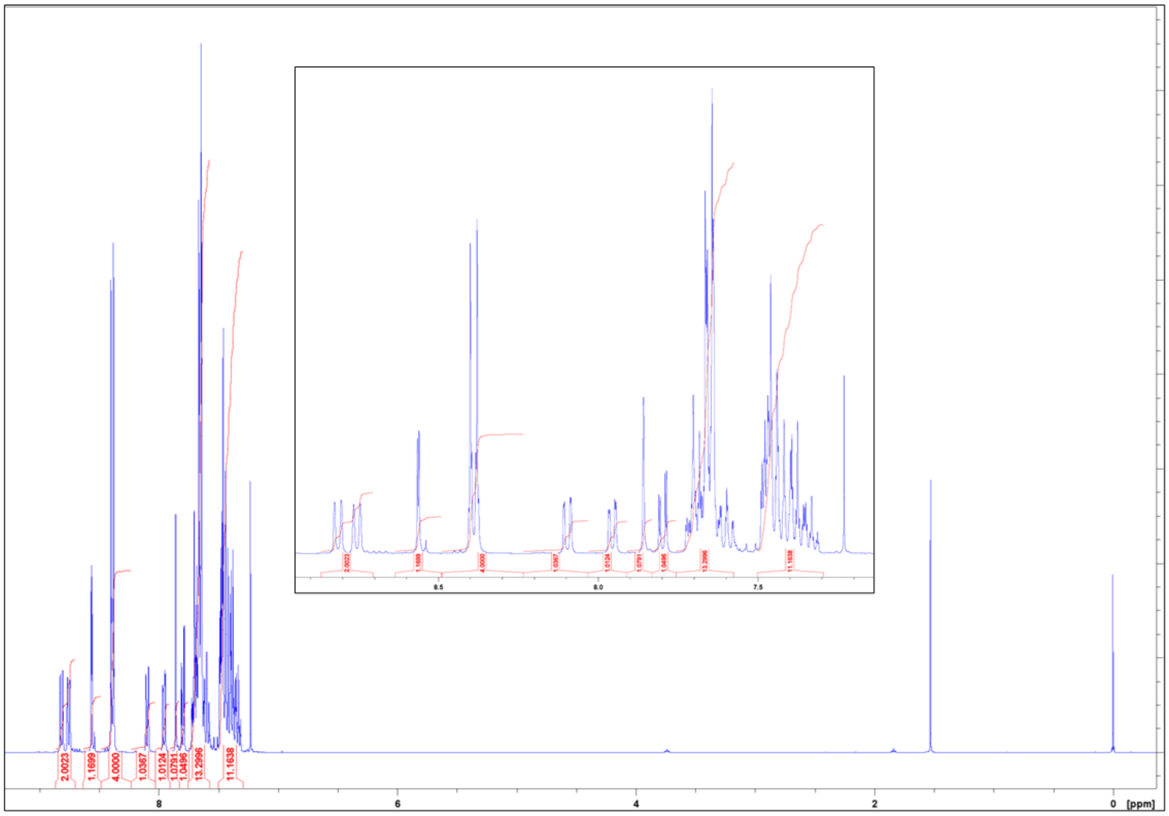

2.0023
1.1699
4.0000
1.0367
1.0124
1.0791
1.0496
13.2996
11.1638
8
6
4
2
0
[ppm]

(j)

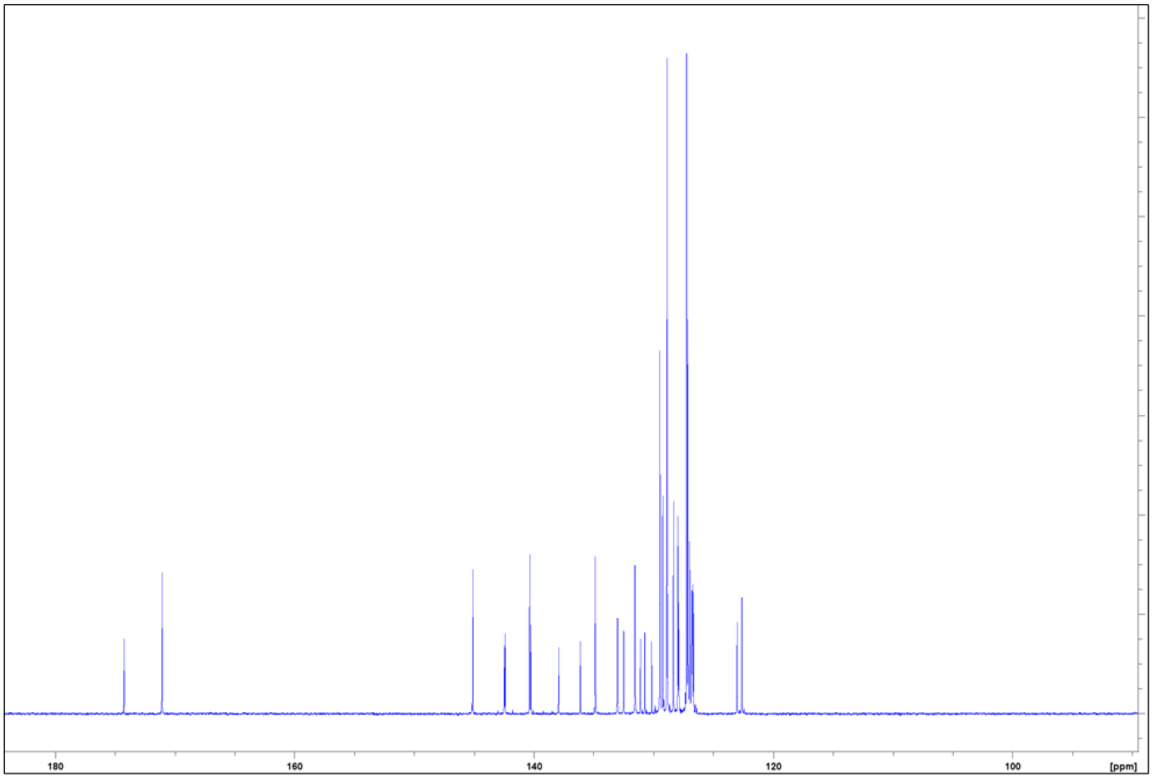

180
160
140
120
100
[ppm]

(k)

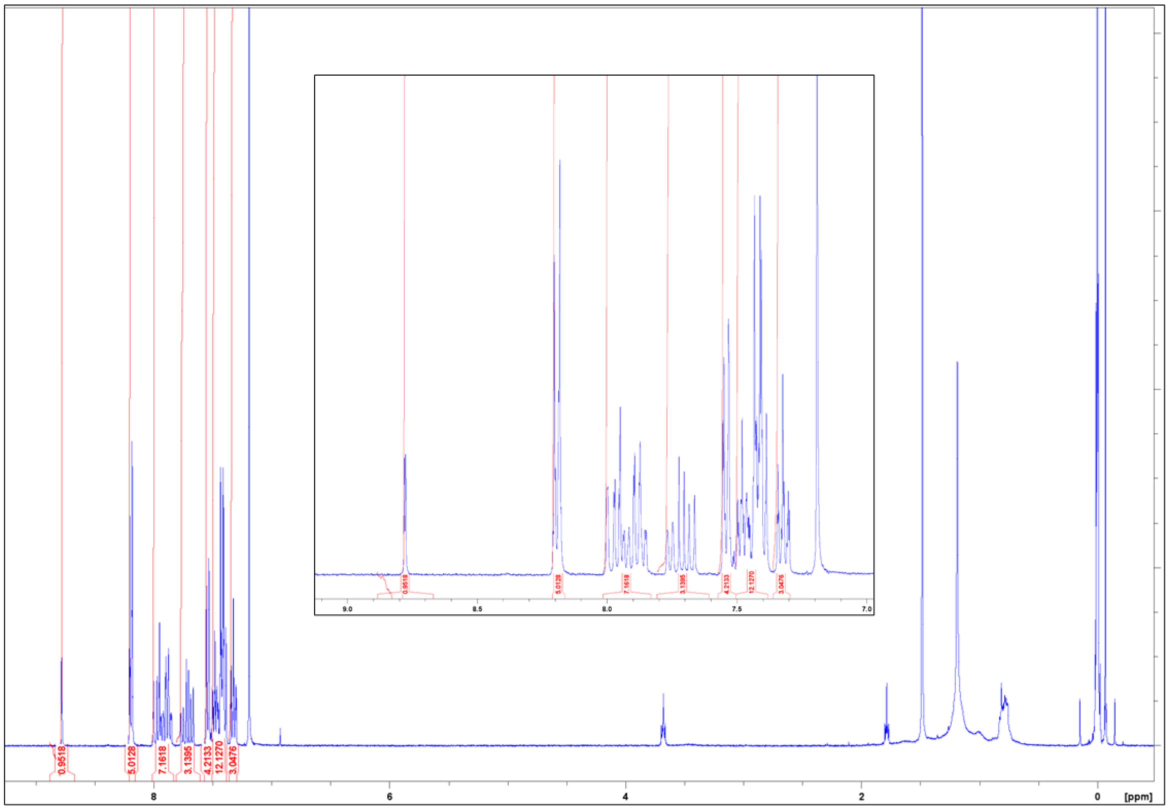
0.9518
5.0128
7.1618
3.1395
4.2133
12.1270
3.0476
8
6
4
2
0
[ppm]
9.0
8.5
8.0
7.5
7.0

(l)

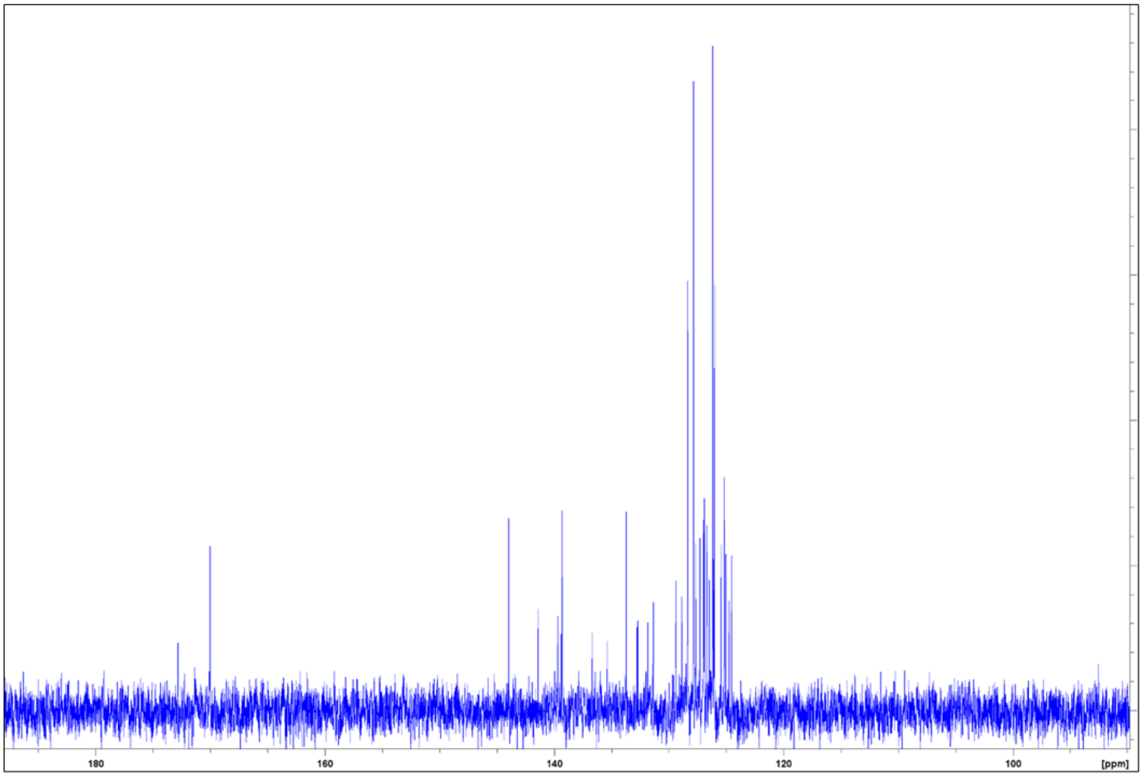
180
160
140
120
100
[ppm]

(m)

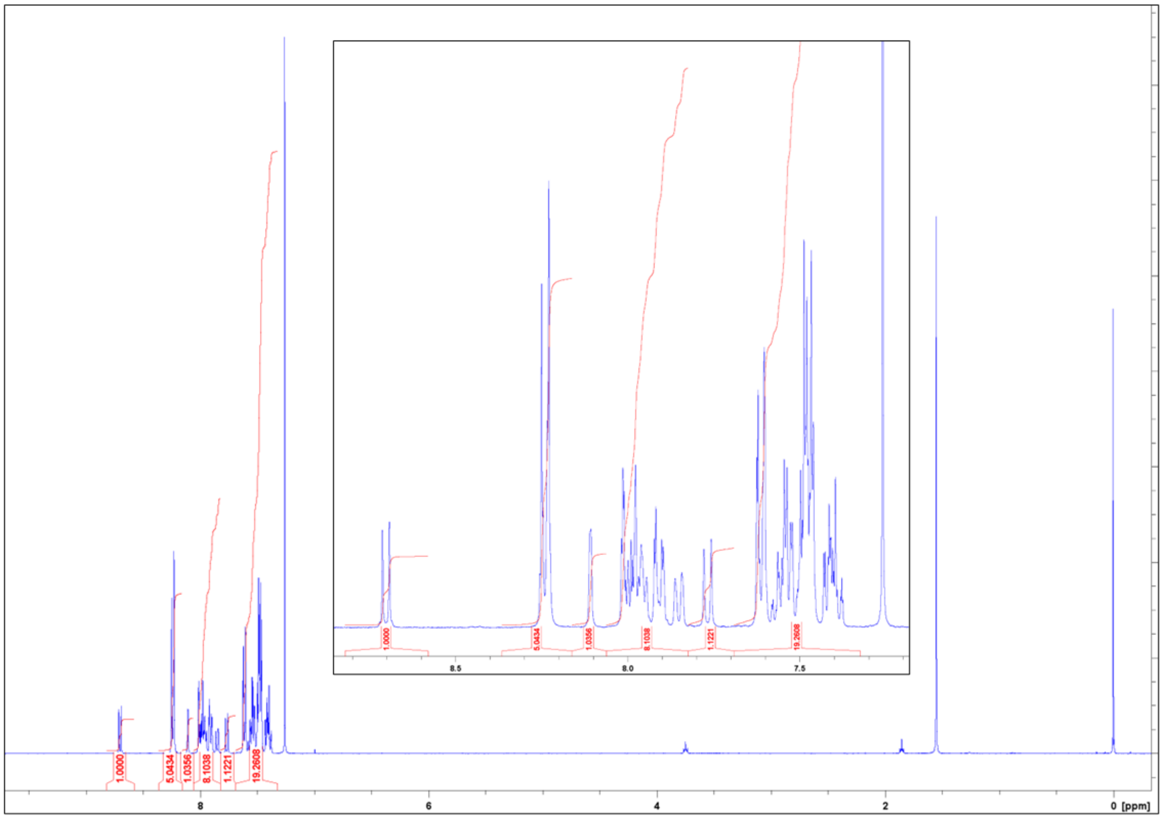
1.0000
5.0434
1.0356
8.1038
1.1221
19.2608
8
6
4
2
0 [ppm]
8.5
8.0
7.5

(n)

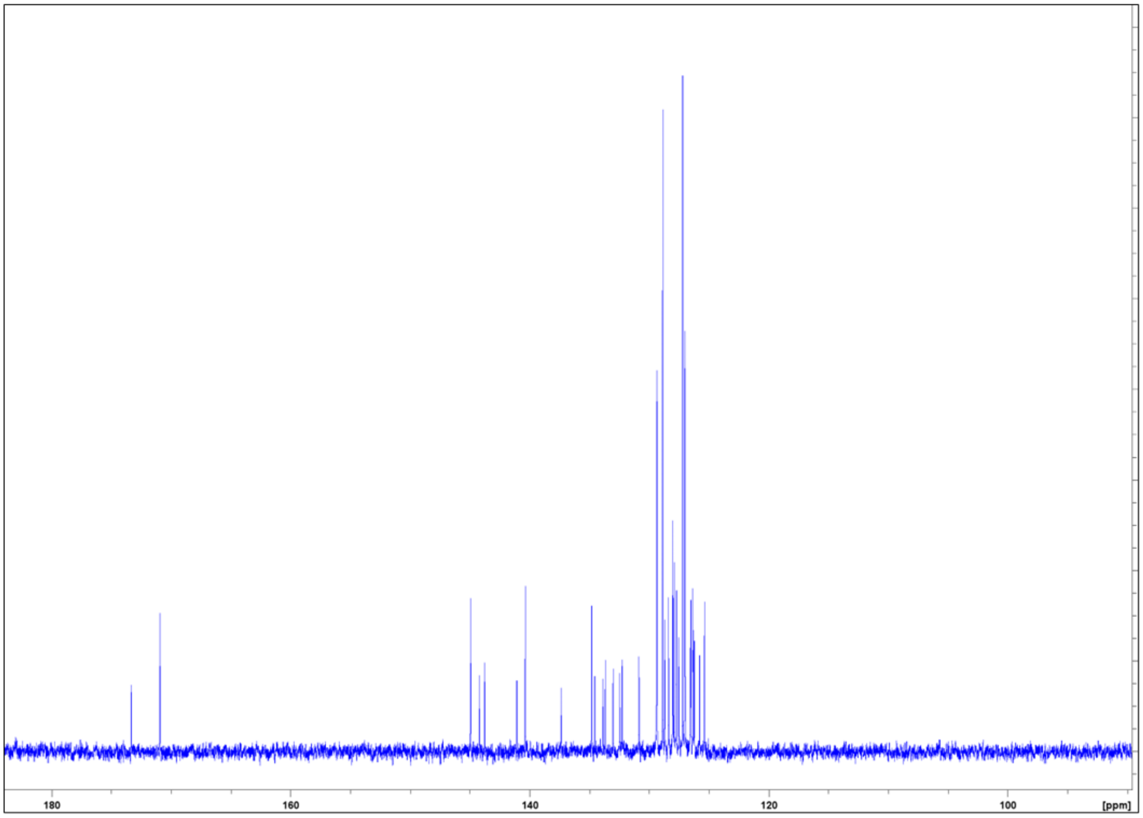
180
160
140
120
100
[ppm]

(o)

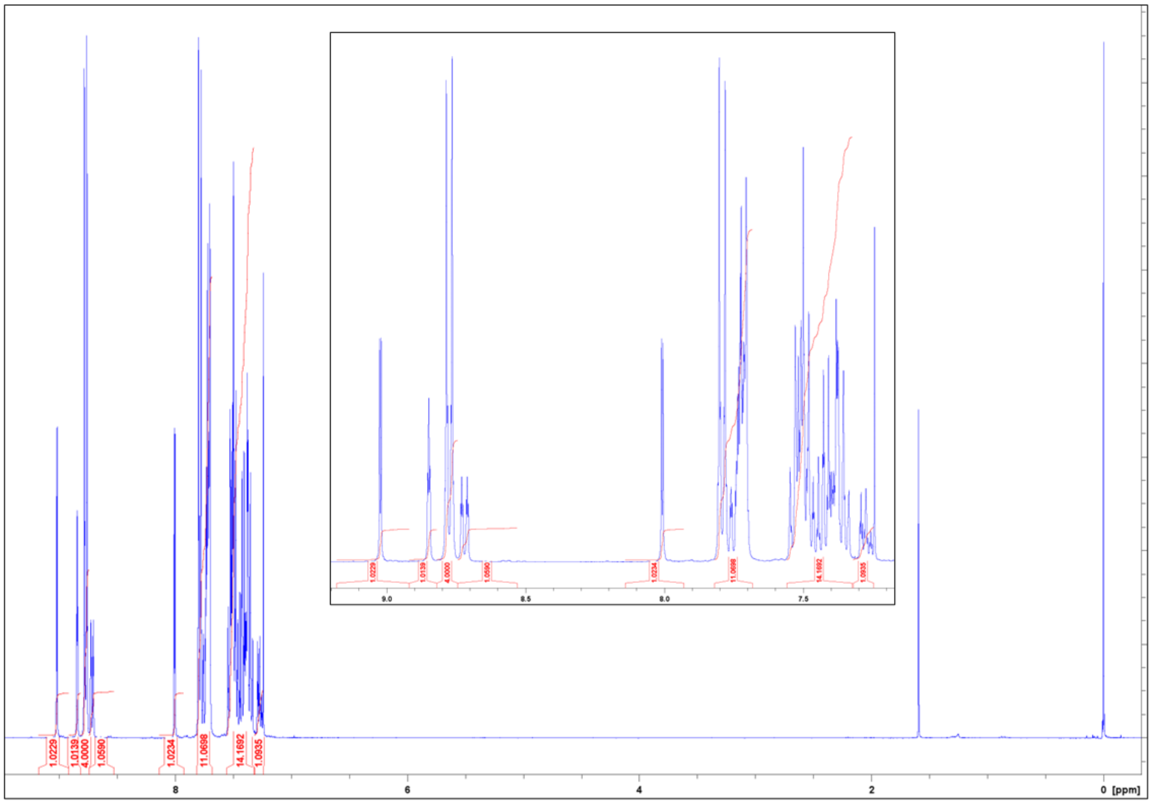
1.0229
1.0139
4.0000
1.0590
1.0234
11.0698
14.1692
1.0935
9.0
8.5
8.0
7.5
8
6
4
2
0 [ppm]

(p)

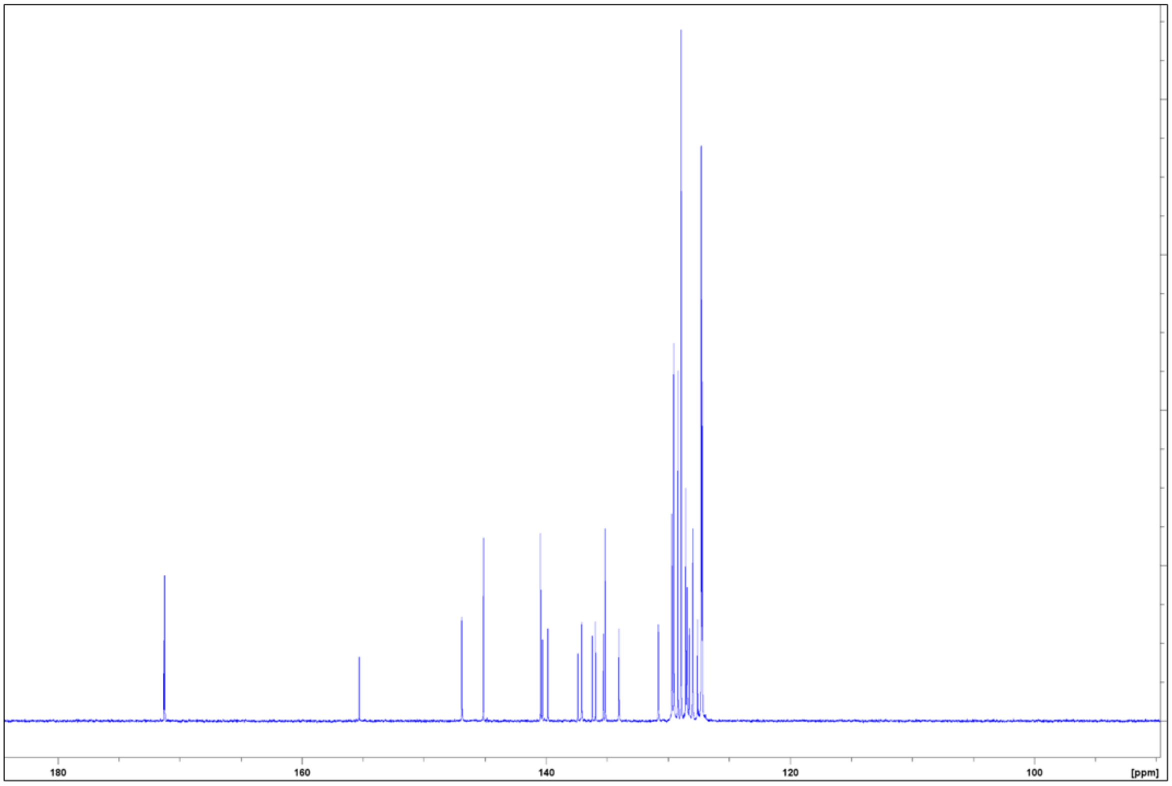
180
160
140
120
100
[ppm]

(q)

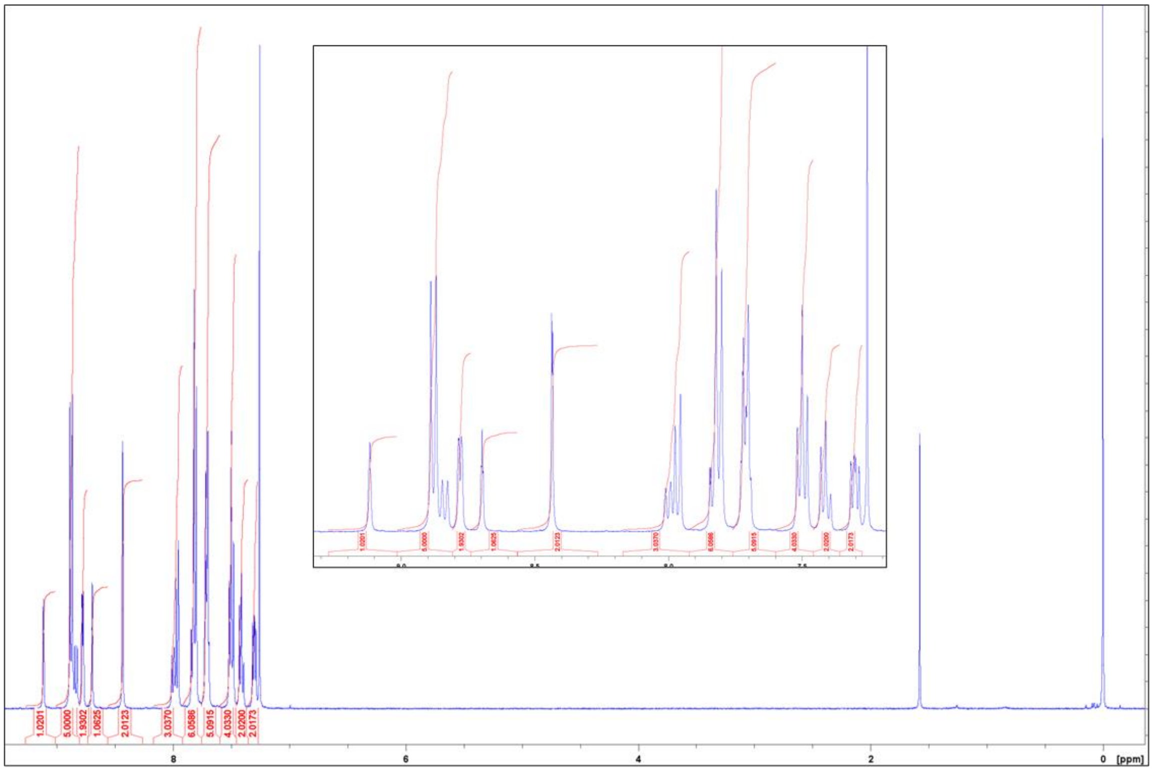

1.0201
5.0000
1.9302
1.0625
2.0123
3.0370
6.0586
5.0915
4.0330
2.0200
2.0173
8
6
4
2
0
[ppm]

(r)

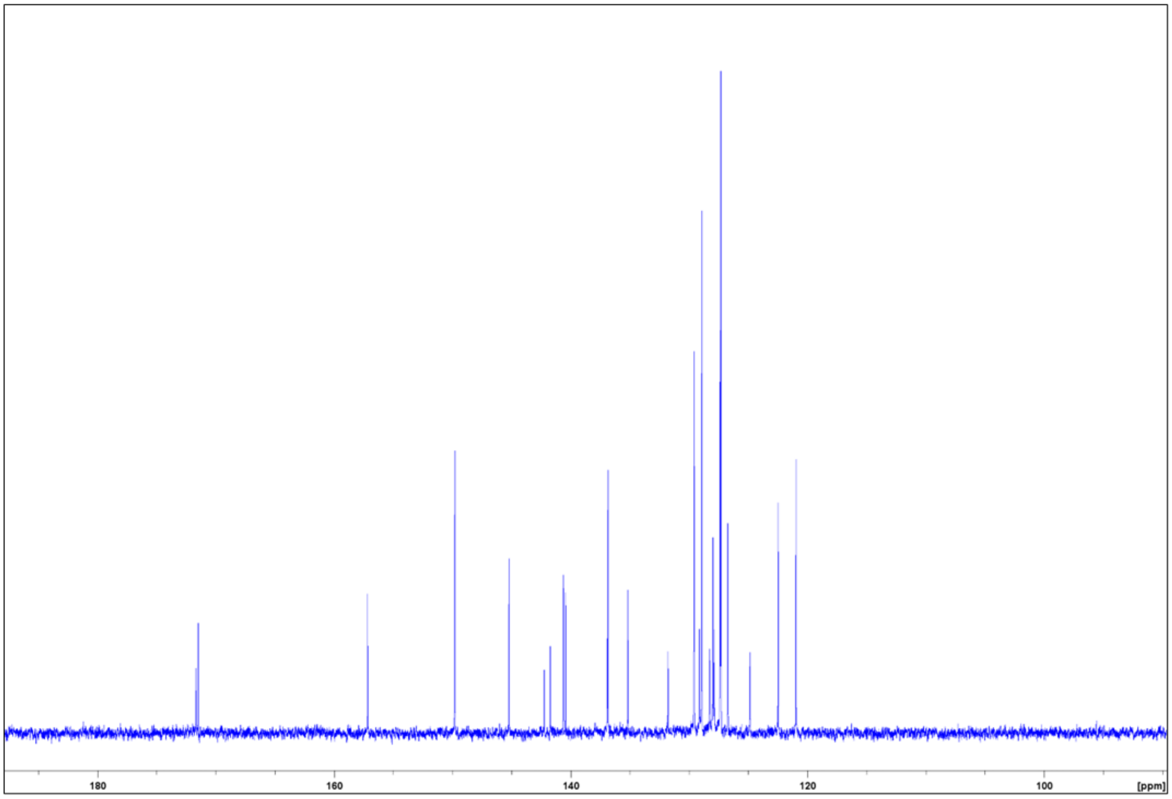

180
160
140
120
100
[ppm]

Figure S5 ^{1}H NMR charts of (a)**T2-7668**, (c)**T2-6104**, (e)**T2-6970**, (g)**T2-7191**, (i)**T4-442**, (k)**T4-2766**, (m)**T3-3317**, (o)**T1-4799**, (q)**T1-5248**
and $^{13}C\{^{1}H\}$ NMR charts of (b)**T2-7668**, (d)**T2-6104**, (f)**T2-6970**, (h)**T2-7191**, (j)**T4-442**, (l)**T4-2766**, (n)**T3-3317**, (p)**T1-4799**, (r)**T1-5248**

(a)

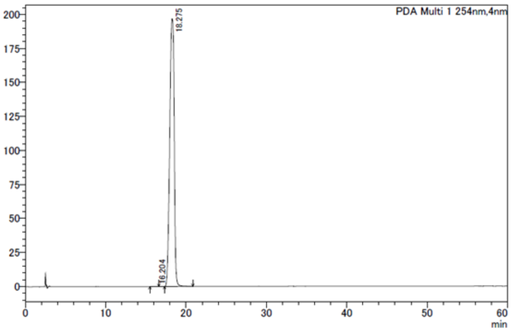

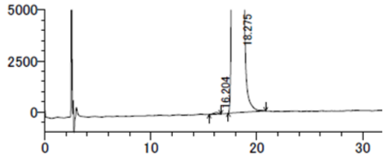

	Retention time / min	Peak area / a.u.	Peak height / a.u.	Area / %
1	16.204	2484	78	0.032
2	18.275	7666016	196848	99.968

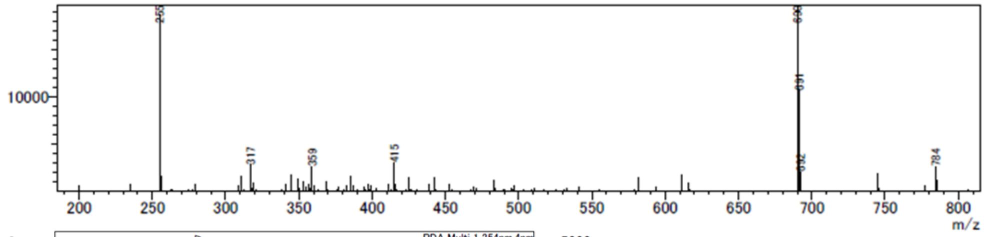

(b)

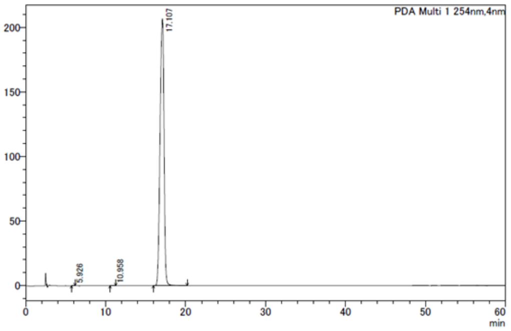

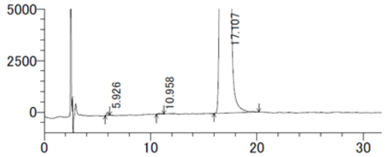

	Retention time / min	Peak area / a.u.	Peak height / a.u.	Area / %
1	5.926	1522	139	0.021
2	10.958	720	36	0.010
3	17.107	7147433	206617	99.969

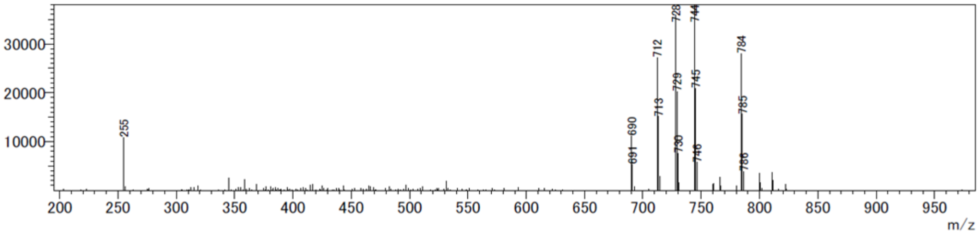

(c)

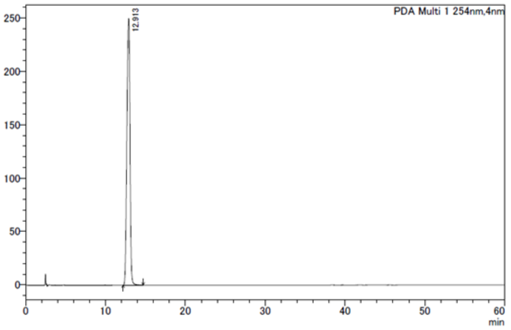

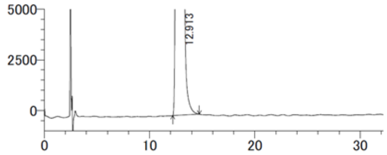

	Retention time / min	Peak area / a.u.	Peak height / a.u.	Area / %
1	12.913	6778729	249662	100.000

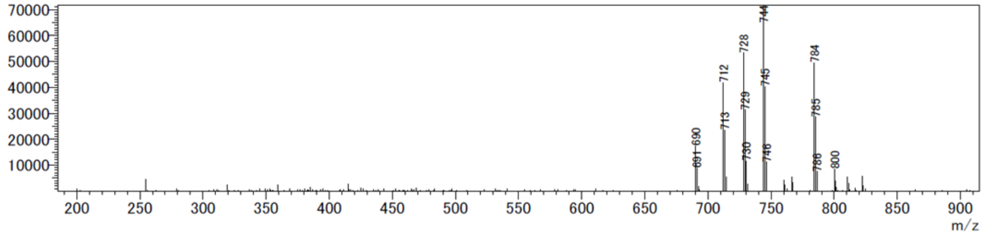

(d)

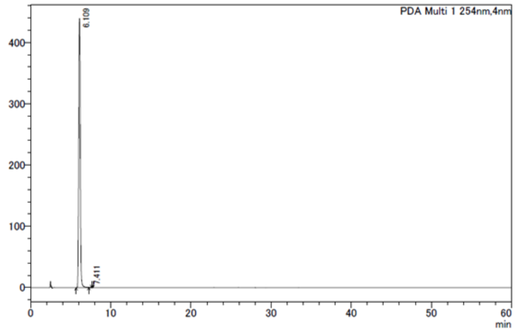

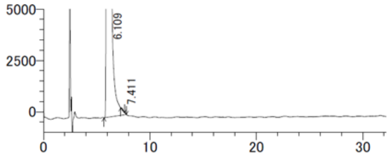

	Retention time / min	Peak area / a.u.	Peak height / a.u.	Area / %
1	6.109	5955953	439343	99.987
2	7.411	752	76	0.013

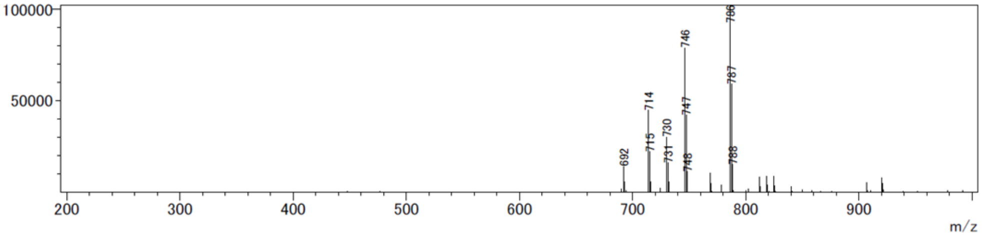

(e)

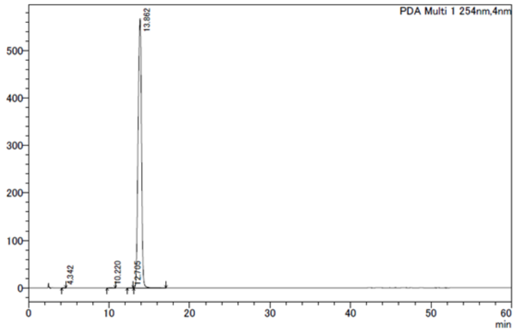

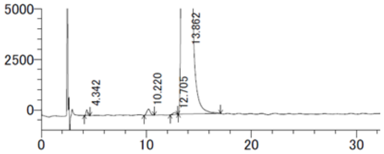

	Retention time / min	Peak area / a.u.	Peak height / a.u.	Area / %
1	4.342	2816	282	0.017
2	10.220	6776	307	0.040
3	12.705	2216	101	0.013
4	13.862	16745203	568351	99.930

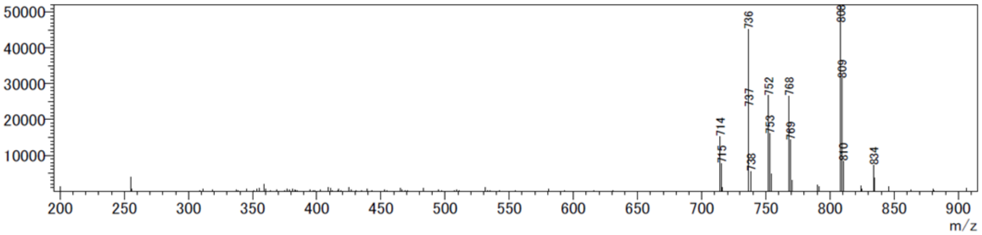

(f)

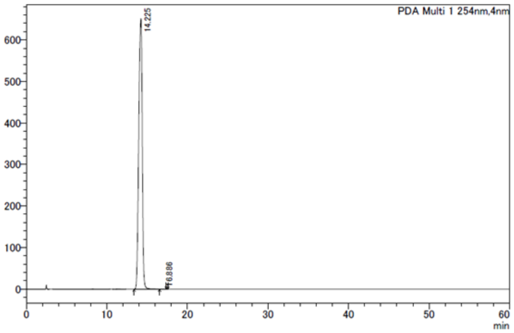

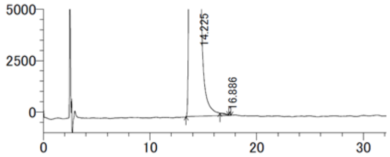

	Retention time / min	Peak area / a.u.	Peak height / a.u.	Area / %
1	14.225	19775959	651623	99.996
2	16.886	793	31	0.004

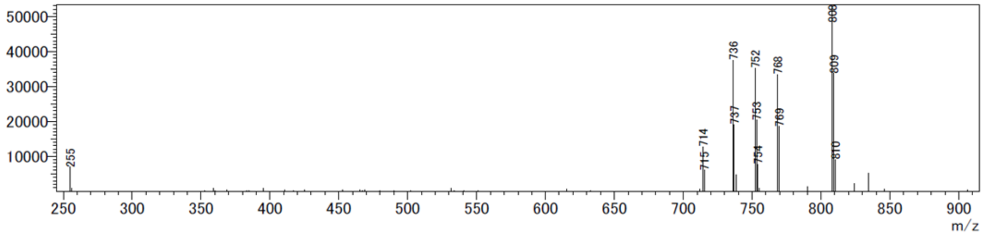

(g)

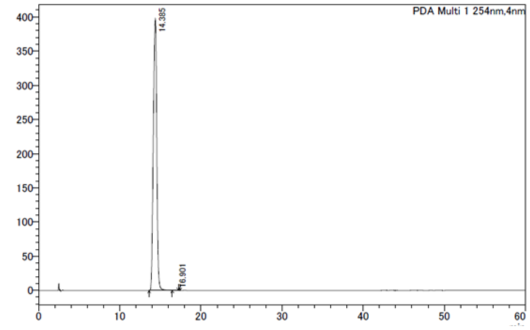

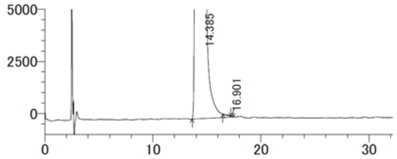

	Retention time / min	Peak area / a.u.	Peak height / a.u.	Area / %
1	14.385	11900609	397968	99.996
2	16.901	492	23	0.004

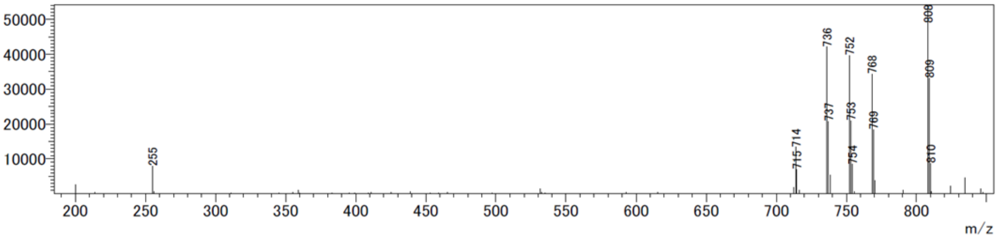

(h)

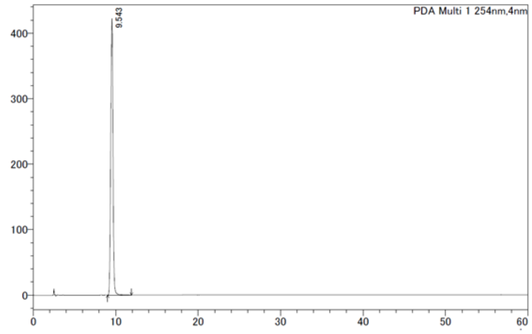

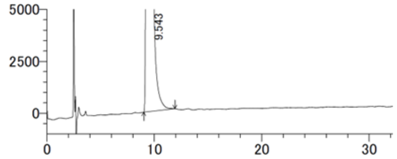

	Retention time / min	Peak area / a.u.	Peak height / a.u.	Area / %
1	9.543	8231602	422128	100.000

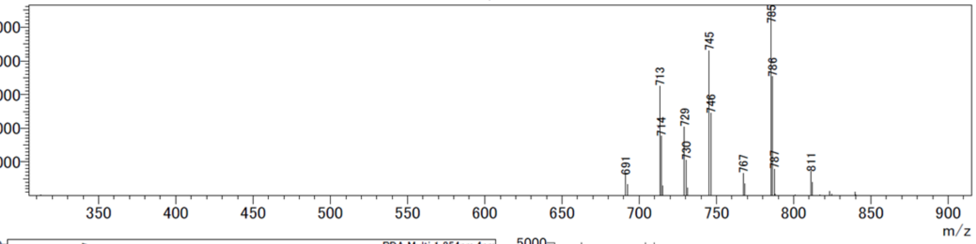

(i)

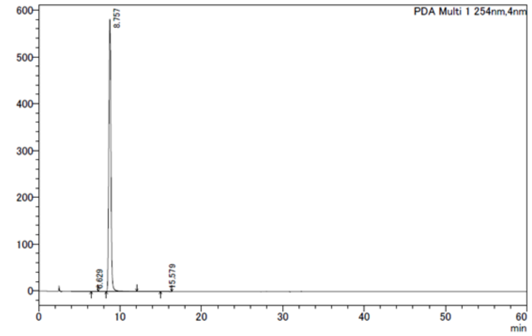

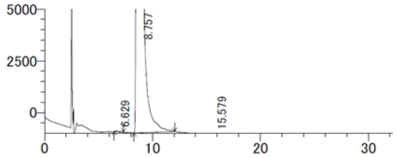

	Retention time / min	Peak area / a.u.	Peak height / a.u.	Area / %
1	6.629	1146	45	0.011
2	8.757	10448882	582025	99.967
3	15.579	2267	67	0.022

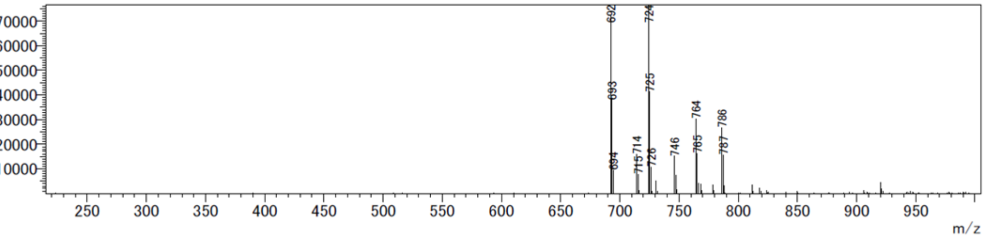

Figure S3 Raw chromatogram, magnified chromatogram, the corresponding peak table, and MS profile of the synthesized samples. (a)**T2-7668**, (b)**T2-6104**, (c)**T2-6970**, (d)**T2-7191**, (e)**T4-442**, (f)**T4-2766**, (g)**T3-3317**, (h)**T1-4799**, (i)**T1-5248**

Supporting references

[s1] TOSOH corporation and Sagami Chemical Research Institute: Uehara, F., Yamagata, T., Nishiura, T., Sato, K., Aihara, H., “Azine Compound, Organic Electroluminescent Element Material Containing Said Azine Compound, And Organic Electroluminescent Element”, WO2022/025142A1

[s2] TOSOH corporation: Uehara, F., Hattori, K., Takahashi, Y., Shono, T., Uchida, N., ”Triazine Compound, Material for Organic Electroluminescent Elements, Electron Transport Material for Organic Electroluminescent Elements, And Organic Electroluminescent Element”, WO2022/075219 A1

[s3] Jilin University: Wang, Y., Wu, Y., Li, Y., Liu, Y., Lu, D., Shen, J., “(Hydroxyphenyl)pyridine derivative, its metal complexes and application as electroluminescence material”, CN1245822

[s4] TOSOH corporation: Ono, Y., Oike, K., Uehara, F., Takahashi, Y., JP2022157926A

[s5] TOSOH corporation and Sagami Chemical Research Institute: Arai, N., Sato, K., Hamaguchi, N., Hirano, M., Aihara, H., Yamagata, T., Ono, Y., Shinya, H., Kawashima, H., Nomura, K., JP 2021102601 A

[s6] Avitia, B., MacIntosh, E., Muhia, S. & Kelson, E. Single-flask preparation of polyazatriaryl ligands by sequential borylation/Suzuki–Miyaura coupling. *Tetrahedron Lett.* **52**, 1631–1634 (2011).

<Previous studies about T_g prediction>

[s7] Kim, Y. S., Kim, J. H., Kim, J. S. & No, K. T. Prediction of glass transition temperature (Tg) of some compounds in organic electroluminescent devices with their molecular properties. *J. Chem. Inf. Comp. Sci.* **42**, 75–81 (2002).

[s8] Yin, S., Shuai, Z. & Wang, Y. A Quantitative structure−property relationship study of the glass transition temperature of OLED materials. *J. Chem. Inf. Comp. Sci.* **43**, 970–977 (2003).

[s9] Xu, J. & Chen, B. Prediction of glass transition temperatures of OLED materials using topological indices. *J. Mol. Model.* **12**, 24–33 (2005).

[s10] Barbosa-da-Silva, R. & Stefani, R. QSPR based on support vector machines to predict the glass

transition temperature of compounds used in manufacturing OLEDs. *Mol. Simulat.* **39**, 234–244 (2013).

[s11] Zhao, Y., Fu, C., Fu, L., Liu Y., Lu, Z. & Pu, X. Data-driven machine learning models for quick prediction of thermal stability properties of OLED materials. *Mater. Today Chem.* **22**, 100625 (2021).

[s12] Kwak, H. S. *et al.* Design of Organic Electronic Materials with a Goal-Directed Generative Model Powered by Deep Neural Networks and High-Throughput Molecular Simulations. *Front. Chem.* **9**, 800370 (2022).
